# Transcriptome and Network Changes in Climbers at Extreme Altitudes

**DOI:** 10.1371/journal.pone.0031645

**Published:** 2012-02-29

**Authors:** Fang Chen, Wei Zhang, Yu Liang, Jialiang Huang, Kui Li, Christopher D. Green, Jiancheng Liu, Guojie Zhang, Bing Zhou, Xin Yi, Wei Wang, Hang Liu, Xiaohong Xu, Feng Shen, Ning Qu, Yading Wang, Guoyi Gao, A. San, LuoSang JiangBai, Hua Sang, Xiangdong Fang, Karsten Kristiansen, Huanming Yang, Jun Wang, Jing-Dong J. Han, Jian Wang

**Affiliations:** 1 BGI Shenzhen, Shenzhen, China; 2 Chinese Academy of Sciences Key Laboratory of Computational Biology, CAS-MPG Partner Institute for Computational Biology, Chinese Academy of Sciences, Shanghai, China; 3 Beijing Institute of Genomics, Chinese Academy of Sciences, Beijing, China; 4 Center for Molecular Systems Biology, Institute of Genetics and Developmental Biology, Chinese Academy of Sciences, Beijing, China; 5 The Graduate University of Chinese Academy of Sciences, Beijing, China; 6 The Hospital of XiShuangBanNa Dai Nationalities Autonomous, Jinghong, China; 7 Department of Biology, University of Copenhagen, Copenhagen, Denmark; The Centre for Research and Technology, Hellas, Greece

## Abstract

Extreme altitude can induce a range of cellular and systemic responses. Although it is known that hypoxia underlies the major changes and that the physiological responses include hemodynamic changes and erythropoiesis, the molecular mechanisms and signaling pathways mediating such changes are largely unknown. To obtain a more complete picture of the transcriptional regulatory landscape and networks involved in extreme altitude response, we followed four climbers on an expedition up Mount Xixiabangma (8,012 m), and collected blood samples at four stages during the climb for mRNA and miRNA expression assays. By analyzing dynamic changes of gene networks in response to extreme altitudes, we uncovered a highly modular network with 7 modules of various functions that changed in response to extreme altitudes. The erythrocyte differentiation module is the most prominently up-regulated, reflecting increased erythrocyte differentiation from hematopoietic stem cells, probably at the expense of differentiation into other cell lineages. These changes are accompanied by coordinated down-regulation of general translation. Network topology and flow analyses also uncovered regulators known to modulate hypoxia responses and erythrocyte development, as well as unknown regulators, such as the OCT4 gene, an important regulator in stem cells and assumed to only function in stem cells. We predicted computationally and validated experimentally that increased OCT4 expression at extreme altitude can directly elevate the expression of hemoglobin genes. Our approach established a new framework for analyzing the transcriptional regulatory network from a very limited number of samples.

## Introduction

At extreme altitudes (>5500 m above sea level) the partial pressure of oxygen is less than a half of the sea level. At these conditions, humans experience hypobaric hypoxia, which can induce a range of normal or adverse responses at cellular and systemic levels. At the cellular level, there is increased expression of genes that participate in anaerobic energy supply and decreased expression of those involved in ATP consumption processes [1]. At the systems level, the major physiological responses include increased heart and ventilation rate and rapid erythrocyte expansion [2], [3]. Normally, symptoms of increased heart rate and hyperventilation would appear within the first few hours when ascending to high-altitude, which is due to stimulation of the peripheral chemoreceptors by hypoxia. In the following days, increased hemoglobin concentration and erythrocyte numbers would be observed in the blood, which is more likely to be a gene-level regulation of the body. These signs together with other response of different systems of the body comprised altitude acclimatization. When maladapted, a person can develop high altitude illnesses that range from mild, acute mountain sickness (AMS), which occurs in 50–90% of the populations, to fatal high-altitude cerebral edema (HACE) and high-altitude pulmonary edema (HAPE), which have much lower frequencies [4], [5]. Field studies on these physiological aspects have generated a great deal of valuable data for understanding these processes, including ECG measurements [6], [7], arterial blood gases, and oxygen content [8]. The molecular mechanisms of hypoxia and high altitude acclimatization are more limited and primarily include information about hypoxia-induced factors (HIFs) and the role of erythropoietin in the high altitude response [1], [2], [3]. The signaling pathways and molecular functions that mediate such responses (especially those that modulate or are independent of HIFs and erythropoietin) are largely unknown.

**Figure 1:**
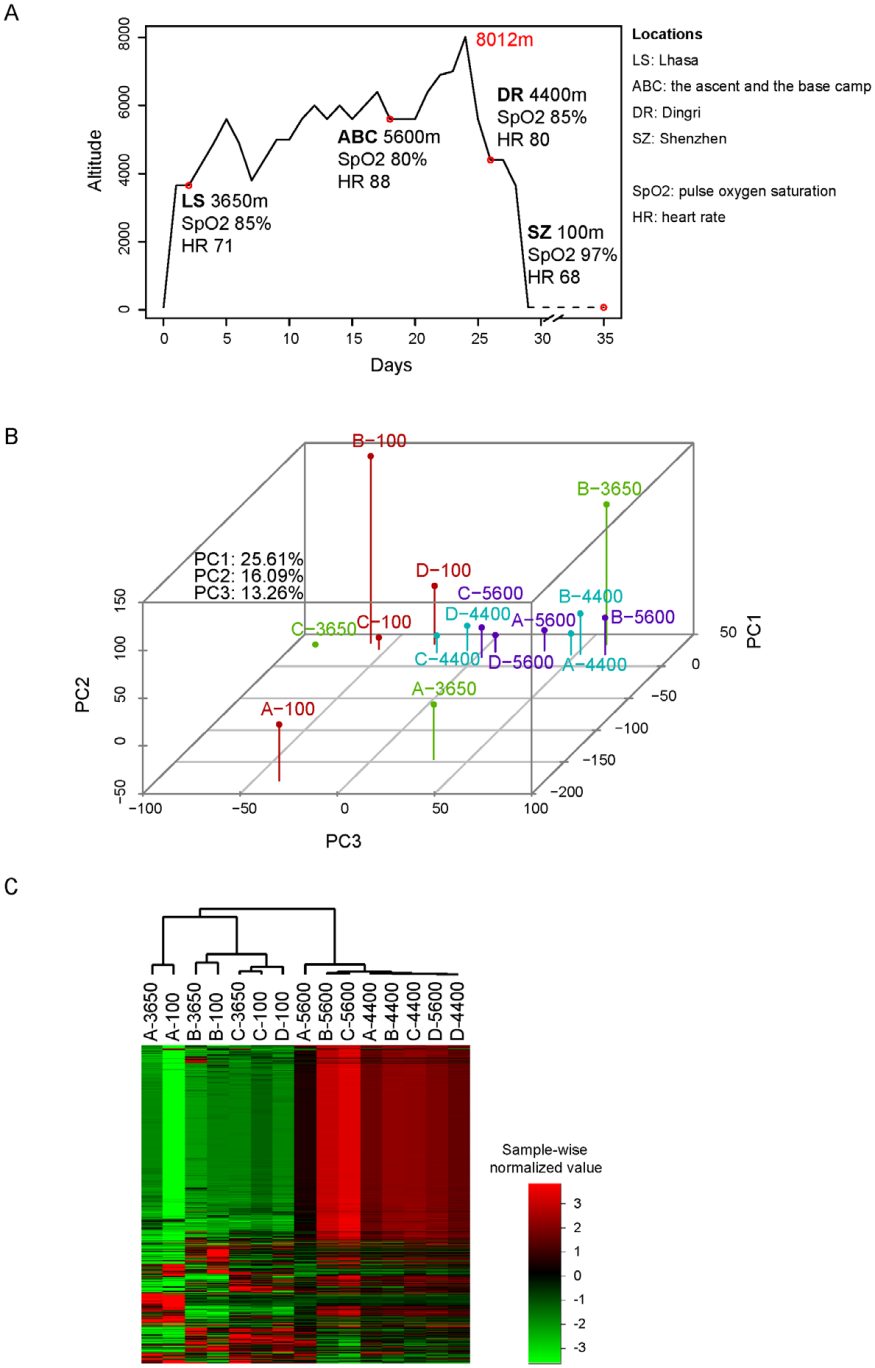
RNA expression pattern changes in samples from climbers at four different altitudes. (**A**) Illustration of the climbing path and the sampling locations with average SpO_2_ and HR of four subjects. (**B**) PCA of all samples based on expression profiles of all genes measured by RNA-seq. The four colors represent the four different altitudes. (**C**) Hierarchical clustering of all samples based on expression profiles of all genes measured by RNA-seq. Samples are named in the format of ‘Climber ID-altitude’. The expression value for each gene is indicated by color intensity, with red representing high expression and green representing low expression.

To obtain a more complete picture of the transcriptional regulatory landscape and networks involved in extreme altitude response, we accompanied four individuals on a climbing expedition and measured the transcriptome (mRNA and miRNA expression levels) using RNA-seq from blood samples taken before, during, and a month after climbing Mount Xixiabangma (Figure 1A). To the best of our knowledge there has been so far no study on genome-wide expression changes in human high-altitude response and that ours is the first one and is therefore important in shedding lights on this very important human acclimation process and prevention of the pathological effects associated with it.

We chose to collect blood samples mainly because of its easy accessibility, which is extremely important if gene expression changes revealed were to be used as biomarkers for diagnosing maladaptation and pathological response in the future. Meanwhile, we can monitor the transcriptome changes in both nucleated erythrocytes and white blood cells by measuring the blood samples, which is very important as both the increased erythropoiesis and inflammatory response are known to have a close link with hypoxia [2], [3], [9], [10], [11].

Due to the large individual variations and the limited sample size, we developed a novel network-based analysis approach to identify the differentially expressed network modules and important regulators in response to extreme altitude. By doing so, we uncovered a highly modular network with seven modules of various functions, with the erythrocyte differentiation module being the most prominently up-regulated, indicative of shifted differentiation of hematopoietic stem cells to increase erythrocyte fate at the expense of other alternative cell fates. The network modules also revealed a coordinated down-regulation of genes involved in translation elongation at extreme altitudes. Network topology and flow analyses further uncovered both known and novel regulators of hypoxia responses and erythrocyte development.

## Results

### Climbers' information

Four climbers participated in this study, including two ∼55-year-old males (A and B, A is a frequent climber of Mount Xixiabangma), a 44-year-old, well-trained male mountaineer (C), and a 33-year-old female (D). The four individuals did not travel above 3,000 m in the past six month, and they did not suffer from any severe altitude sicknesses after they arrived in Lhasa and during the mountain-climbing. Blood samples were taken at Lhasa (LS, 3,650 m above sea level) during the ascent and the base camp (ABC, >5600 m), at Dingri (DR, 4,400 m), during the descent, and at Shenzhen (SZ, 100 m) a month later (Figure 1A, Methods).

### General profiles of transcriptome in response to extreme altitudes

Principal Component Analysis (PCA) of the data showed there was large variance within the expression profiles of different individuals at lower altitudes (100 m and 3,650 m), especially for the older individuals, but at higher altitudes (5,600 m and 4,400 m) the expression profiles of different individuals look quite similar (Figure 1B). Hierarchical clustering of the samples clearly separated the profiles into two major clusters according to the high (4,400 m and 5,600 m) and low altitudes (100 m and 3,650 m) (Figure 1C, Figure S1A), indicating extreme altitude induces a common transcriptional regulatory program. Moreover, within the 100 m and 3650 m sample cluster, the expression profiles grouped by individual rather than by altitude, whereas those at extreme altitudes were more uniform, indicative of decreased individual variances at extreme altitudes (Figure 1C). Climber C as a well-trained mountain climber had low-altitude profiles that were closest to the clustering seen in the extreme-altitude profiles (Figure 1B).

Although >3000 m is typically defined as being of high altitude, the expression profiles we obtained at 3,650 m from the climbers were more similar to those at 100 m than those at 4,400 m and 5,600 m. These profiles, however, do not necessarily indicate the environmental factors of the 3,650 m are more similar to the sea level (100 m) than those of the two higher altitudes (4,400 m and 5,600 m). In fact, it may be very likely due to the climbers' lack of acclimatization time at 3,650 m (1 day) compared with 5,600 m and 4,400 m which have more than 15 days exposure at high altitude (Figure 1A), and/or due to the climbers' intense physical activities (climbing) at 4,400 m and 5,600 m compared with 100 m or 3,650 m.

It should also be noticed that the observed differential gene expression might not be merely caused by the environmental factors at different altitudes, e.g. the partial pressure of oxygen, the amount of exposure to the ultraviolet light, the temperature, etc. It could also be caused by other factors of the climbers, e.g. different food, intense physical activities, dehydration, etc. Due to the limitation of our not fully controlled experiment design, which is difficult to achieve given the extreme nature of the environment studied, we could not exclude the possibility of other factors and suggest the high altitude alone has a causal role for the differential expression.

We performed the RankProd package [12] to identify differentially expressed genes (DEGs). In total, 723 DEGs including 380 up- and 343 down-regulated genes were found in response to extreme altitudes (Methods, Table S1). Then we identified the enriched Gene Ontology (GO) terms [13] and Kyoto Encyclopedia of Genes and Genomes (KEGG) pathways [14] in response to extreme altitude using the Fisher's exact test and Gene Set Enrichment Analysis (GSEA) [15] (Methods). We found that the most significantly up-regulated gene sets are “hemoglobin complex”, “inflammatory response”, “cytoskeleton”, “myeloid cell differentiation”, and “oxidoreductase activity”, whereas the most significantly down-regulated ones are “ribosome”, “platelet alpha granule lumen”, and “killing of cells of another organism” (Table S2, Table S3, Table S4 and Figure S2).

### Modular network responsive to extreme altitudes

To obtain robust analysis results, we based our analysis at the level of functional interactions, which are defined as the union of the interactions from 3 sources: KEGG [14], STRING [16] and IntNetDB [17] (Supplementary Methods S1). First, we constructed an altitude-responsive network (hereafter referred to as the ARN for short) that includes 310 genes among the 723 DEGs (Methods, Table S1, Figure S3A). To further distinguish the functions that are up- or down-regulated, we separated the ARN into 172 up-regulated genes with 341 edges and 89 down-regulated genes with 250 edges in the up- and down-regulated networks, respectively (Figure S3B).

**Figure 2:**
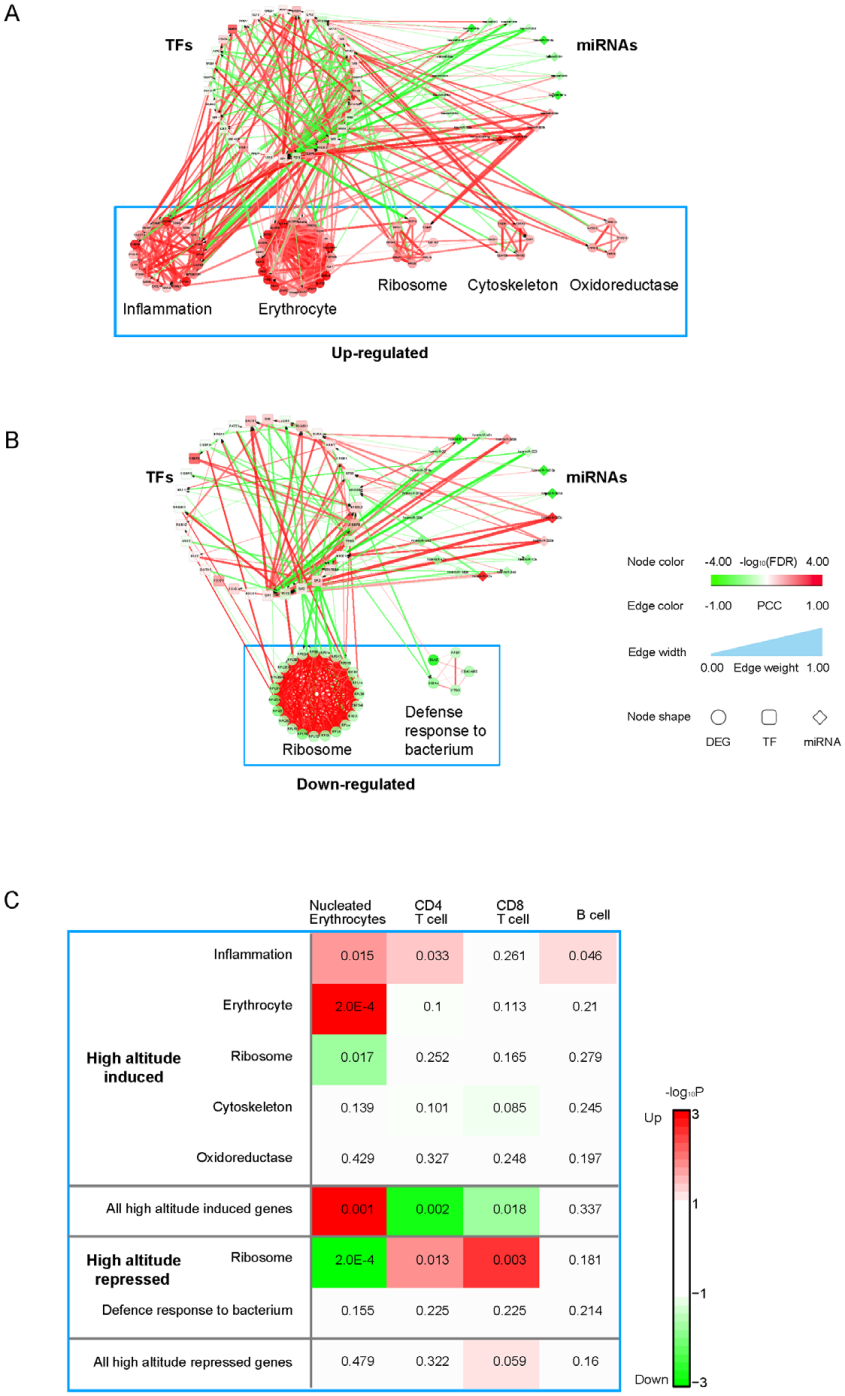
Modular network reponse to extreme altitude. (**A** and **B**) The regulatory networks for up- (**A**) or down-regulated (**B**) gene expression at extreme altitude as compared to low altitudes illustrating the number of genes, gene density, function, regulations and structures of the modules and structures of the modular organization of the networks. TFs are visualized in the upper left circle, miRNAs in the upper right circle, and network modules in the up- or down-regulated altitude-responsive networks are each visualized as a circle. The color intensity of a node denotes the weight of the node, with red representing a positive weight value and up-regulation, and green representing a negative weight value and down-regulation. The width of an edge indicates the weight of the edge. The color of an edge indicates the expression correlation between the two genes connected by the edge. All other network graphs are similarly annotated except for the ResponseNet (Figure 3B). Graphic keys include node size, edge width, node and edge colors and shapes. (**C**) Expression level changes of up- and down-regulated network modules when differentiated hematopoietic cell lineages are compared to hematopoietic stem cells (HSCs).

Visualization of the up- and down-regulated networks revealed the obvious existence of network modules (clusters of tightly connected subnetworks) in each of the networks (Figure S3B, Table S5). Due to the high density of interactions in ARN, the modules could hardly be manually separated. However, they could be clearly delineated using a Markov Clustering algorithm (Methods, Figure 2A and B).

By examining enriched GO terms and KEGG pathways using the Fisher's exact test, we found that the up-regulated network contained five modules corresponding to the functions ‘inflammation’, ‘erythrocyte development/oxygen binding’, ‘ribosome’, ‘cytoskeleton’, and ‘oxdoreductase’, respectively. In the down-regulated network, we found two modules that were enriched for the functions ‘ribosome’ and ‘defense response to bacterium’, respectively (Figure 2A and B, Table S6, Table S7). These modules' functions were very similar to the findings of all the DEGs (Table S2, Table S3, Table S4 and Figure S2). Although the ribosome genes appeared in both an up-regulated module and a down-regulated module, the down-regulated ribosome module is much larger than the up-regulated one (23 versus 8 genes, respectively, Figure 2A and B), indicating the translation elongation is generally decreased in response to extreme altitude. This is accompanied with the increase of erythrocyte and inflammatory genes which are known to be related with high altitude response and hypoxia [1], [2], [3], [9], [10], [11]. Thus, various genes routinely expressed in different individuals are turned off or down at the translation level and the gene expression landscape is reshaped toward those necessary to respond to hypoxia and other extreme altitude induced stress. This might explain the reduction in the gene expression variation among different individuals at extreme altitudes (Figure 1).

We wondered whether this reflected the increased erythrocyte differentiation from the hematopoietic stem cells at the expense of differentiation into other cell lineages in response to hypoxia and/or extreme altitudes, in which case we should see genes that are preferentially expressed in erythrocytes up-regulated, whereas those preferentially expressed in other cell lineages repressed at extreme altitudes. We examined a published gene expression dataset [18] on four differentiated hematopoietic cell lineages and their parental hematopoietic stem cells (HSCs). Indeed, genes that are specifically expressed in erythrocytes are significantly up-regulated (*P* = 4.31×10^−2^) and those expressed in CD8+ cells are significantly repressed (*P* = 2.84×10^−3^) at extreme altitudes (methods). In addition, looking at mRNA obtained from different cell types, we found that the extreme-altitude-induced genes are significantly up-regulated in erythrocytes and down-regulated in T cells, whereas the suppressed genes are moderately up-regulated in the CD8+ T cells compared to HSCs (Figure 2C). In particular, when compared to the HSCs, genes in the ‘erythrocyte development/oxygen binding’ module are significantly up-regulated in nucleated erythrocytes, whereas those in the down-regulated ‘ribosome’ module were significantly up-regulated in the CD4+ and CD8+ T cell lineages, but down-regulated in erythrocytes (Figure 2C). This finding is of particular interest as decreased immunity has been reported as a common response to high altitudes [19].

### Identifying transcriptional regulation of altitude response

**Figure 3:**
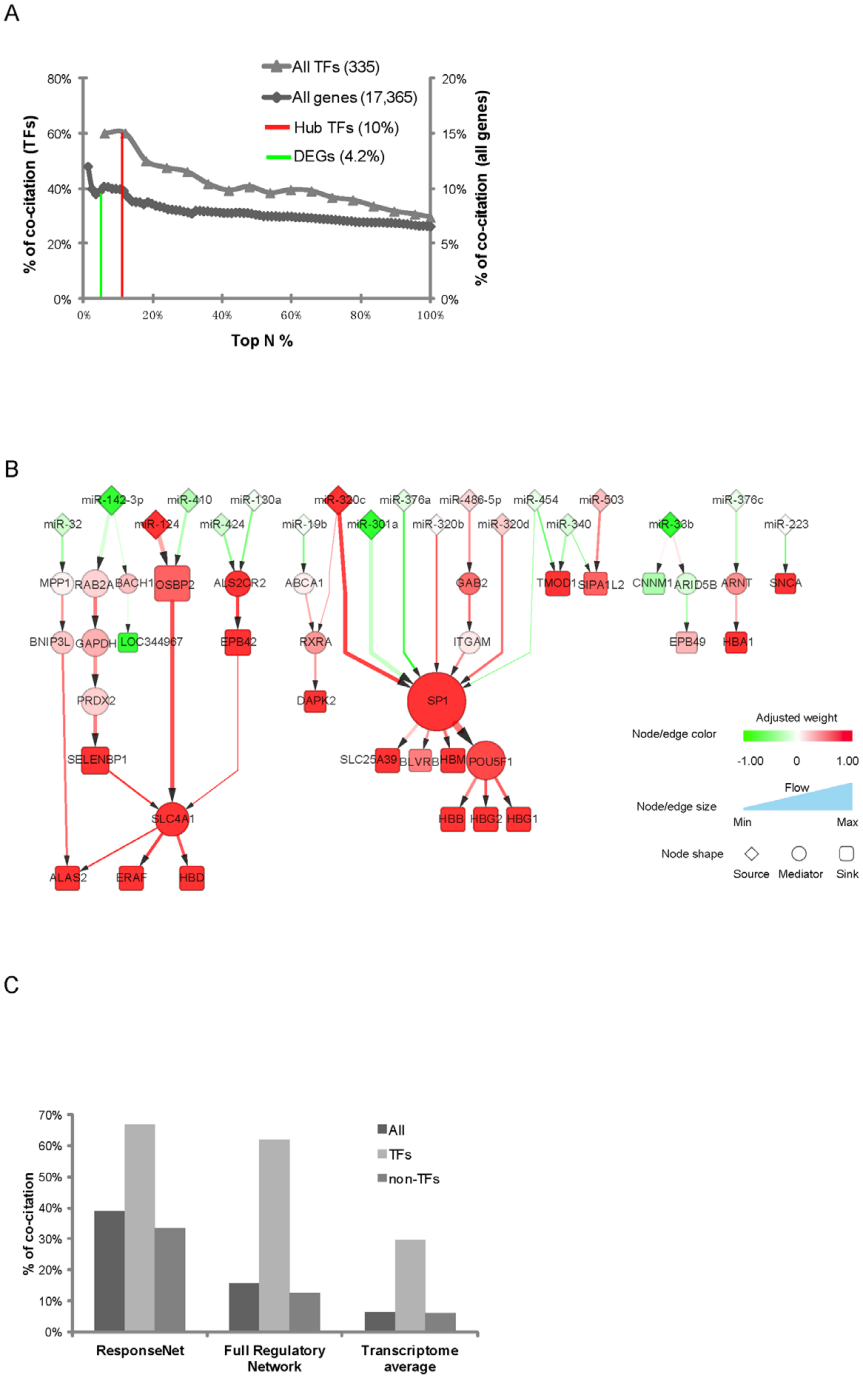
Nodes and pathways important to extreme-altitude response. (**A**) The frequency of literature co-citation with keywords “hypoxia”, “hypoxic”, and “altitude”is plotted for the top N percent of all 335 TFs (triangles) that have motif position weight matrix (PWM) in TRANSFAC or JASPAR database (left y-axis), or of all 17,365 genes (diamonds) detected by mRNA-seq (right y-axis) ranked by their nodes weight (x-axis). The 723 DEGs and the 34 hub TFs are marked by the red and green vertical lines, respectively. (**B**) ResponseNet from differentially expressed miRNAs to the genes unanimously up- or down-regulated at extreme altitude. Node and edge colors are annotated as the scaled weights. Node size and edge width are proportional to the amount of flow passing through a node or an edge. (**C**) The frequency of the literature co-citation among genes in the ResponseNet compared to those in the full regulatory network and the whole transcriptome.

To find the key transcriptional regulators of the ARN, we first examined whether the weight of nodes (Table S1) in the ARN was an indicator of the degree of association of the genes with ‘hypoxia’ or ‘high-altitude’. We found that the frequency of literature co-citation of a gene with these keywords increased with the weight of a node in the network (Figure 3A). Then to find potential transcriptional regulators giving rise to the differential gene expression networks, we searched for potential transcription factors (TFs) of the DEGs and differentially expressed miRNAs (Supplementary Methods S1).

We evaluated the relatedness of the TFs to the ARN by weighting the TFs to reflect their prestige centrality [20] (Supplementary Methods S1). Although the weights of TFs did not require the TFs themselves to be significantly up- or down-regulated at extreme altitudes, they still correlated well with the frequency of literature co-citation with hypoxia or extreme-altitude (Figure 3A). When the TFs were similarly weighted as the DEGs according to the significance of their own expression changes, the weight of the TFs is less correlated with the frequency of literature co-citation (data not shown). The overall co-citation frequency (Figure 3A) and the average number of co-citation times (Table S1) of the top 10% weighted TFs (here after referred to as hub TFs and were used for the further analysis) were 6.6 and 20.4 times those of the DEGs, indicating these hub TFs we found through the network analysis are more likely to be mechanistically responsible for the extreme-altitude response than are the DEGs. This is consistent with the notion that transcription factors do not need to be significantly up- or down-regulated in order to induce significant downstream gene expression changes, and that a network-based search for transcriptional regulators of a complex trait is more powerful than simple differential gene expression analysis.

As miRNA suppression of target genes is generally dependent on a stoichiometrically high dosage of miRNA expression, we defined and weighted differentially expressed miRNAs similarly to the coding genes (Supplementary Methods S1). All the functional interactions in the ARN together with the potential regulatory interactions constitute the altitude regulatory network (hereafter referred to as the Full Regulatory Network or FRN for short) (Methods, Table S8). Such a network allows us to find important network motifs, regulatory nodes, and pathways that mediate the altitude response.

### Key regulators and their action in the altitude-responsive network

The correlation between the percentage of co-citation and the TFs' weights indicate that the weights do reflect the importance of the TFs in the extreme altitude response. We therefore examined the hub TFs (Table S1).

OCT4 (encoded by the *POU5F1* gene), one of the most important factors for stem cell reprogramming), SP1/2/3, TCF3, CEBPB, and HIF1B (ARNT), had the most significant weights and were thus predicted to be the most important transcription regulators of the network. The weights of these TFs are consistent with their central positions in the regulatory networks (Figure 2A and B). The POU5F1 gene itself was also 4.48 fold up-regulated at extreme altitude among the four people (RankProd *P* = 0.0012). In the regulatory network, it appears to be activated by SP1/2 and regulate the erythrocyte differentiation/oxygen binding network module through transcriptional activation of hemoglobin gamma 2 (HBG2) and hemoglobin beta (HBB) (Figure S4B).

For the miRNAs, miR-629, miR-1308, miR-124 and miR320c were the most concordantly up-regulated, and miR-33b, miR-301a, miR-142-3p, miR-21* and miR-487b were the most concordantly down-regulated at extreme altitude. With both a large number of targets and a high average weight of target genes, miR-124, miR-320b/c/d, miR-340 and miR-142-3p seem to be important regulators of both the up- and down-regulatory networks (Figure 2A and B). We also found that the miRNAs predominantly target the TFs rather than the DEGs, indicating they may regulate genes' expression indirectly in response to extreme altitude (Figure 2A and B).

### Pathways responding to extreme altitudes

As shown in Figure 2A and B, even using very stringent cutoffs to search for potential regulators of the gene expression changes in response to high altitude, we are still left with too many of such possible regulatory TFs and miRNAs. Therefore, to globally and unbiasedly identify which of the regulators are the most critical ones for further experimental validations, we employed an flow optimization algorithm called ResponseNet [21] (Methods).

This analysis captured the significantly high ranked nodes and delineated their potential molecular relationships in interactive pathways (Figure 3B and Table S9). In total, 6 out of the 34 hub TFs (17.6%) and 30 out of 501 (6.0%) non-TF DEGs from the FRN are included (Table S9). In addition, the TFs of very high weights, especially SP1 and POU5F1, were indeed high-flow nodes passing information down to the essential set of only 20 genes commonly up- or down-regulated in all four individuals (the sink nodes, shown in the graph as terminal nodes at the bottom of the hierarchy) (Figure 3B).

**Figure 4:**
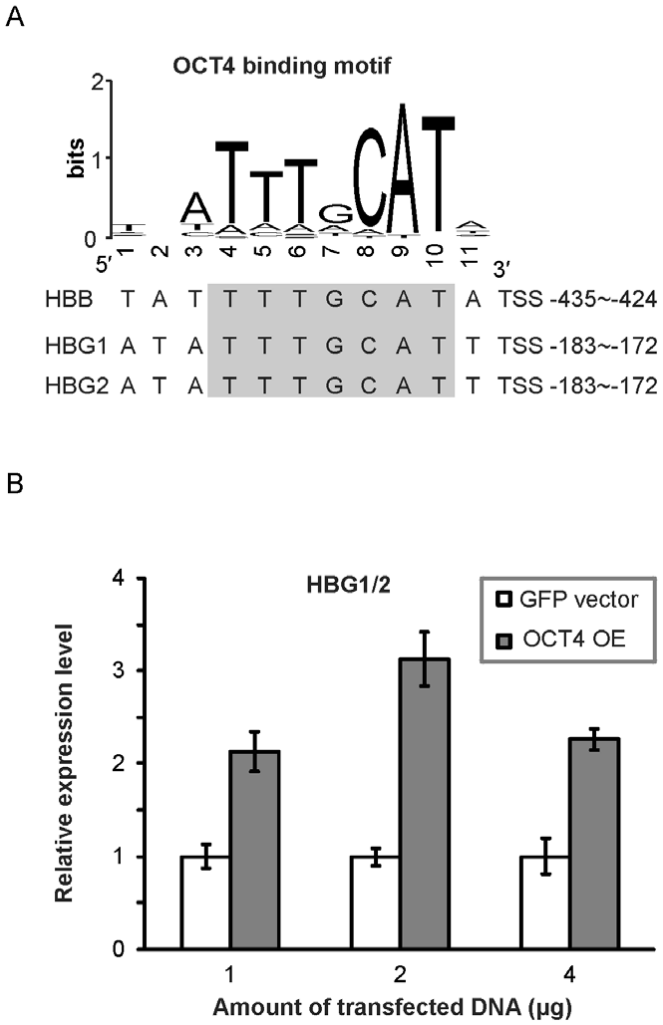
OCT4 binding sites and regulatoion of HBB, HBG1, and HBG2. (**A**) The OCT4 binding motif logo shows the position weight matrix of the OCT4 in the TRANSFAC database. The predicted OCT4 binding sites on HBB, HBG1, and HBG2 are listed for comparison to the censensus motif. (**B**) Fold change of HBG1 and 2 (HBG1/2) mRNA expression level upon OCT4 overexpression. HeLa cells were transfected with 1, 2 and 4 µg of control GFP-containing vector or OCT4, each time three wells of cultured cells were measured separately by qPCR. Error bars represent standard deviations (marked by the wiskers). OE standands for overexpression.

As a depiction outlining pathways with the most important information flow, nodes in this highly distilled flow network showed the overall highest rate of co-citation with hypoxia or altitude, even when compared to the rest of the FRN (Figure 3C). The nodes in these pathways are primarily regulators that are known to be induced by hypoxia and to mediate hypoxia responses, such as SP1 and HIF1B (ARNT), or are important to erythrocyte development, such as ALAS2 and ERAF. The ResponseNet further delineated potential pathways that were formed by these genes to mediate the extreme altitude response. Of interest, this analysis also pinpointed previously unsuspected regulators, such as OCT4 (encoded by *POU5F1*), onto these pathways. The identification of these provides a more complete and unbiased map of the high-altitude response pathways. Our analysis predicted that OCT4 is downstream of SP1 and upstream of hemoglobins HBB, HBG1, and HBG2 (Figure 3B). As OCT4 is normally lowly expressed and is assumed to have no function in differentiated cells, it is very surprising that OCT4 was predicted by our model to increase the expression of hemoglobins HBB, HBG1, and HBG2 and hence promote erythrocyte development. We therefore visually confirmed the existence of the OCT4 binding motifs on these genes (Figure 4A) and tried to verify these regulatory relationships experimentally. By overexpressing the OCT4 gene in HeLa cells, we found that the level of transcripts for HBG1 and 2, which are not distinguishable and denoted HBG1/2, were indeed significantly up-regulated 2- to 3-fold compared to vector-transfected cells (Figure 4B).

## Discussion

Our data and analyses revealed that at extreme altitudes the most significant up-regulation occurred for the erythrocyte development module and the inflammation module whereas coordinated down-regulation occurred for modules involved in translation elongation. This might reflect a shift of hematopoietic stem cell differentiation towards erythrocyte production at the expense of other differentiated cell lineages. Our regulatory network analysis identified feedback loops involving both miRNA and TFs that were potential mechanisms for altering network homeostasis (Figure S5). Pathway analysis by flow optimization further revealed regulators that were previously known to be induced by hypoxia and to mediate hypoxia responses, including SP1 and HIF1B (ARNT), or important to erythrocyte development, such as ALAS2 and ERAF. We were also able to identify regulators that had not previously been suspected as important for these response networks, such as OCT4 (POU5F1), and validated the predicted regulation of γ-globin by OCT4.

Previous studies indicated OCT4, which is best known for its critical role in regulating embryonic stem cell pluripotency, is expressed in somatic stem cells (including HSC and other bone marrow derived stem cells) but not in somatic tissues [22]. Since we measured the transcripts in whole blood, our finding of OCT4 up-regulation might be due to an increase in the number of HSCs or erythroid progenitor cells being released into peripheral blood circulation under extreme altitude. Given the very small fraction of HSC in the whole blood, we cannot formally exclude this possibility despite that the HSC specific genes identified by Chambers et al. [18] were either not changed or significantly down-regulated at high altitude in the four different climbers (data not shown). HIF-2α (EPAS1) has been shown to be able to increase OCT4 expression under hypoxia [23], and certain variants of HIF-2α have been found by several recent studies to be associated with high-altitude adaptation and low hemoglobin concentration in Tibetans [24], [25], [26], [27]. According to Chambers et al's data [18], HIF-2α also expresses in nucleated erythrocytes. It should be noted that the activation of HIFs by hypoxia is not through the induction of their gene expression but through posttranslational modifications on the encoded proteins [23]. This may explain the lack of HIF-2α in our network.

Although our data in this study showed that the gene expression profiles of different individuals become more similar at extreme altitude, given their large initial variation at low altitudes, some of the changes in gene expression between low and extreme altitudes must be non-homogeneous. Indeed, we found that even the most concordantly changed functional gene sets identified by GSEA sometimes varied in the extent of changes (Figure S2). As a case in point, the well-trained young climber (C), who has many times of 8000-meters climbing experiences, showed the greatest deviation from the consensus changes (Figure S2). For instance, unlike the other three climbers, climber C showed no gene expression changes in the myeloid cell differentiation and cytoskeleton, and only displayed moderate up-regulation of inflammatory response, which is shown to have a close relationship with hypoxia and underlies the pathogenesis of high-altitude pulmonary edema (HAPE) [9], [10], [11] (Figure S2). It indicates that these functions, especially inflammatory response, might reflect secondary responses that could probably be avoided through training and/or experiences of mountaineering.

It remains unclear what changes occur in white blood cells at high altitude [28], and, due to the limitations imposed by the environment, we did not perform a blood cell count. However, the fact that we extracted less total RNA in samples taken at extreme altitudes may indicate a decrease in the number of white blood cells as RNA from blood samples are primarily derived from white blood cells with our experimental procedure. This would be consistent with our proposal that there is a compensatory decrease of white blood cells due to the developmental shift of HSCs to an erythrocyte cell fate. Given the impact of changes in blood composition and compensatory activity, more detailed assessment of the impact of high altitude on blood cell development would be of interest for future study.

Climbing an 8,000-meter-high mountain is an arduous human endeavor by itself, let alone performing blood sampling and processing under such primitive conditions, including the need to carry the necessary equipment (e.g. a blood centrifuge) up the mountain. Thus, the samples we could collect were limited. Yet our gene expression and network analysis on the obtained peripheral blood of only four individuals of different age, sex, and mountaineering experience still revealed a common highly organized transcriptional regulation program under extreme altitude despite individual variance. We think our analysis can efficiently return network modules and regulatory pathways because we used many noise reduction procedures: 1) our networks were constructed based not only on the significance of over/under-expression. To remove the noise due to variations given by the limited number of individuals, we first restricted the analysis to only functionally related molecular interactions from several high-confidence interaction databases, and then searched for significantly co-interacting/tight connected and co-regulated modules to focus on functional modules. 2) The predicted regulatory functions were again based on not only the knowledge of TF binding motif, but also the interaction strength between TF and target genes, the significance of target gene differential expression and the total number of putative differentially regulated target genes and so on. All these, as demonstrated by literature co-citation validation, were necessary to reliably retrieve coherent biological information from the noisy data initially thought to be unanalyzable due to the large individual variations unrelated to altitude response, which given the limited sample size cannot be removed by routine multivariate analysis.

In reality, in both clinical practice and routine bench-side experiments, it is hard to obtain hundreds or thousands of samples to do standard association tests. Even then it is not easy to interpret the molecular network underneath a biological response or phenotype. To meaningfully interpret biological networks from a few samples is therefore even more challenging. So far, there is no effective analysis framework available to tackle this problem. Although in order to move the analysis from bench to bedside to realize the goal of personal medicine, such a problem must be addressed. The concordance of our analysis results, their consistency with the current literature and the experimental validation of the predicted OCT4 regulation on γ-globin gene expression fully demonstrated the power of the new analysis approach we established and provided a much needed analysis framework for inferring molecular networks from a very limited number of samples.

## Materials and Methods

### Ethics statements

The research protocol was approved by the Ethics Committee of BGI Shenzhen and written informed consent was obtained from each climber.

### Sample preparation

We collected 10 ml blood samples from four individuals at four different locations along the climb to and descent from Mount Xixiabangma (8,012 m above sea level). In total, we obtained four samples for each climber A, B, and C, and three samples for climber D (no sample was taken for this individual in Lhasa (3,650 m) because she had a cold). We measured the transcripts in the nucleated cells of the peripheral blood. Erythrocytes were immediately removed with erythrocyte lysis buffer (Tris-NH_4_Cl). Then the samples were homogenized in Trizol reagents and frozen in liquid nitrogen. The blood sampling was processed within 20 minutes. To avoid other factors that may affect the blood gene expression, such as circadian rhythm and physical activity, the blood samples were collected in the morning soon after waking. Meanwhile, we also recorded other physiological indices, including heart rate, blood oxygen saturation, systolic blood pressure, and diastolic blood pressure for each volunteer at each sampling location to ensure the consistency of physiological parameters at the time of sample collections (Table S10).

### RNA sequencing and data processing

RNA sequencing and data processing are finished in BGI ShenZhen and performed according to Digital Gene Expression Pipeline of BGI ShenZhen. Detailed methodology was described in Supplementary Methods S1 (Figure S6, Table S11, Table S12, Table S13, Table S14).

### Gene expression analysis

Gene expression values were summed from normalized RNA-seq reads. Hierarchical clustering of gene expression was based on pair-wise distances of *1-PCC* (Pearson correlation coefficient) between gene expression values of each pairing sample with 15 data points, using “average” linkage as for agglomeration.

We compared the extremely high (4,400 m and 5,600 m) and relatively low altitudes (100 m and 3,650 m) to identify differentially expressed genes (DEGs) in response to extreme altitudes. As an approach to identify DEGs, our first attempt using the classical single t-test or ANOVA using all four individuals together turned out to be not appropriate for the data (Supplementary Methods S1). Therefore, we performed the RankProd package [12], which is a non-parametric statistical method to estimate significance levels of genes' differential expression (rank) based on a gene permutation mode. The Benjamini-Hochberg corrected FDR≤0.1 was used as a cutoff to define the DEGs. And we also applied the same method to identify differentially expressed miRNA. More details were provided in Supplementary Methods S1.

For the data of hematopoietic cell lineages [18], RankProd *P*≤0.05 was used as a cutoff to define the specifically expressed genes in a certain differentiated cell lineage compared to HSC.

### GO and KEGG annotation enrichment

Annotation enrichments were calculated as described previously [29]. Basically, the enrichment of GO terms (http://www.geneontology.org/) and KEGG pathways (http://www.genome.jp/kegg/) were calculated by the Fisher's exact test on the 380 up- or 343 down-regulated genes in response to the extreme altitude (Table S2 and S3). The Benjamini-Hochberg corrected FDR≤0.05 was used to determine the enriched functions.

We also performed the gene set enrichment analysis (GSEA) [15] on all the genes detected by the RNA-seq, which were ranked by their RP values given by the RankProd package [12]. A cutoff of FDR≤0.25 was used to define the enriched up- and down-regulated functions (Table S4 and Figure S2).

### Constructing the networks

To construct the altitude-responsive network (ARN), we first searched for functional interactions among the 723 DEGs responsive to the altitude change. We weighted the nodes (genes) to reflect their significance of differential expression, and also developed an edge weighting formula similar to a previously published one [30]. By taking only the most highly weighted interactions between the DEGs (empirical *P*≤0.1), we obtained the ARN that includes 310 genes with 938 functional interactions (Table S1, Figure S3A).

The full regulatory network (FRN) were constructed by simply adding the regulatory interactions (hub TFs and differentially expressed miRNAs to their targets among the DEGs) to the ARN. The nodes and edges were similarly defined despite the TFs were weighted by the prestige centrality [20] to reflect their relatedness to the ARN.

The detailed description of the definitions, formulas, and cutoffs we used to construct the networks were provided in the Supplementary Methods S1.

We also tried to construct a larger ARN based on a less stringent definition of DEGs, which were shown in Supplementary Methods S1 (Figure S7).

### Dissecting structural modules

Structurally dense regions in the networks were first identified by the Markov Clustering (MCL) algorithm (http://www.micans.org/mcl/) with the granularity parameter set to 1.5. Then the nodes with only one edge were removed iteratively until no such nodes existed.

### Literature Co-citation

To calculate literature co-citation of a gene and a term, we first obtained the related PubMed IDs of the query gene ID through the “gene2pubmed” table (ftp://ftp.ncbi.nih.gov/gene/DATA/gene2pubmed.gz) in three species (human, mouse, and rat). Then we searched the abstracts of these PubMed IDs for the co-occurrence of the gene symbol or synonym and the three query terms, which are “hypoxia”, “hypoxic”, and “altitude”. Both the “gene2pubmed” table and abstracts were downloaded from NCBI on 28 Jan, 2010.

**Feedback loops** were found using the breadth-first algorithm.

### Comparing the gene expression of hematopoietic cell lineages

The gene expression data of hematopoietic cell lineages were obtained from Chambers SM *et al.* [18] (GSE6506). Pair-wise Student's t-test was used for network modules and DEGs to determine their significance of differential expression between each differentiated cell lineage and HSC (Figure 2C). And it is also used reversely for each cell lineage's specifically expressed genes to determine their significance of differential expression between high and low altitudes. In either case, replicates of each group were used to calculate a mean for comparison.

### Extracting ResponseNet by network flow

When constructing the ResponseNet, we used miRNAs, which are well-known to be regulatory molecules, as the source nodes, while treating the most concordantly up- and down-regulated genes, those significantly up- or down-regulated in all four climbers (*P*≤0.05), as sink nodes. To reflect the degree of relevance of edges to the altitude response, we defined the cost of an edge as the -log value of the edge weight (Supplementary Methods S1).

We also tried to retain only the anti-correlations between miRNA and targets, which were shown in Supplementary Methods S1 (Figure S8).

### Cell culture and transfection

HeLa cells, obtained from American Type Culture Collection (ATCC), were grown in DMEM with 10% fetal bovine serum (FBS) and seeded in 6-well plates at a concentration of 1×10^6^ cells/well the day before transfection. The OCT4 overexpression plasmid (DpQCXIN-OCT4A-360) and control plasmid (pQCXIN-GFP) used for transfection were kindly provided by Dr. Jianwu Dai [31]. Cells were transfected with 1, 2 or 4 µg of plasmid DNA using Lipofectamine 2000 (Invitrogen) and RNA was extracted 48 hours after transfection. The transfection efficiency was detected by checking the GFP signal through a fluorescence microscope.

### Total RNA extraction and quantitative real-time PCR

Total RNA was extracted from cells using Trizol (Invitrogen) reagent. Reverse transcription was carried out using First Strand cDNA Synthesis Kit (TOYOBO), and then synthesized cDNA was analyzed by real-time quantitative PCR (qPCR) using SYBR Green and a Mx3000P qPCR system (Stratagene). The relative expression changes of the genes were analyzed using the 2^−ΔΔ*C*^_T_ method [32]. Sequences of primers used for qPCR were the following:

OCT4 forward - CGTGAAGCTGGAGAAGGAGAAGCTG,

OCT4 reverse - CAAGGGCCGCAGCTTACACATGTTC;

HBG1/2 forward - CCCAGAGGTTCTTTGACAGC,

HBG1/2 reverse - TTCTCAGGATCCACATGCAG;

GAPDH forward - ACCACAGTCCATGCCATCAC,

GAPDH reverse - TCCACCACCCTGTTGCTGTA. GAPDH was used as internal control.

## Supporting Information

Figure S1 Hierarchical clustering of each individual based on expression profiles of all genes measured by RNA-seq (**A**). Or the Hierarchical clustering of all samples based on expression profiles of differentially expressed genes (DEGs) selected by comparing 100 m vs. 4,400 m and 100 m vs. 5,600 m (**B**), or comparing 3,650 m vs. 4,400 m and 5,600 m (**C**). Samples are named in the format of ‘Climber ID-altitude’. The expression value for each gene is indicated by color intensity, with red representing high expression and green representing low expression.
(TIF)

Figure S2 Gene set enrichment analysis (GSEA) summary of seven up- or down- regulated gene sets (**A**). The expression value represented by -log_10_(Nominal p-val) for each gene set is indicated by color intensity, with red representing high expression and green representing low expression. And enrichment plots of these gene sets in four climbers are provided as a combination or individually (**B**).
(TIF)

Figure S3 The altitude-responsive network (ARN) (**A**) as well as the up- or down-regulated sub-network of the ARN (**B**).
(TIF)

Figure S4 Regulatory networks of the up- or down-regulated network modules. Only the one-step regulatory interactions were included in each module.
(TIF)

Figure S5 Feedback loops of ≤5 steps found in the full regulatory network.
(TIF)

Figure S6 Saturation curves of the samples at the obtained sequencing depth.
(TIF)

Figure S7 The regulatory modular network for up- (**A**) or down-regulated (**B**) genes defined by a less stringent criteria, which is the intersection of at least 2 people with RankProd P-value ≤0.05, and the ResponseNet based on this set of DEGs (**C**).
(TIF)

Figure S8 The ResponseNet of retaining only the anti-correlations between miRNAs and targets. All the other settings are exactly the same as Figure 3B.
(TIF)

Table S1 The full list of differentially expressed genes, TFs, and miRNAs.
(XLS)

Table S2 GO/KEGG enriched in up-regulated genes (FDR< = 0.1, 380 genes).
(XLS)

Table S3 GO/KEGG enriched in down-regulated genes (FDR< = 0.1, 343 genes).
(XLS)

Table S4 Gene Set Enrichment Analysis (GSEA) results (FDR< = 0.25).
(XLS)

Table S5 Topological analysis of the altitude-responsive network (ARN).
(XLS)

Table S6 GO/KEGG enriched in up-regulated modules.
(XLS)

Table S7 GO/KEGG enriched in down-regulated modules.
(XLS)

Table S8 All the functional and regulatory interactions in the Full Regulatory Network.
(XLS)

Table S9 The amount of flows on the nodes in ResponseNet.
(XLS)

Table S10 Physiological indexes for each sample at each sampling location.
(XLS)

Table S11 Data summary of RNA-seq.
(XLS)

Table S12 DGE proflie for each gene.
(XLS)

Table S13 Data summary of miRNA-seq.
(XLS)

Table S14 Expression profiles of microRNA genes.
(XLS)

Methods S1 (DOC)
