# Supplementary figures and images for: Transcriptome and Network Changes in Climbers at Extreme Altitudes
Source: PLoS One. 2012 Feb 29;7(2):e31645. doi: 10.1371/journal.pone.0031645 (PMC3290542; doi:10.1371/journal.pone.0031645)

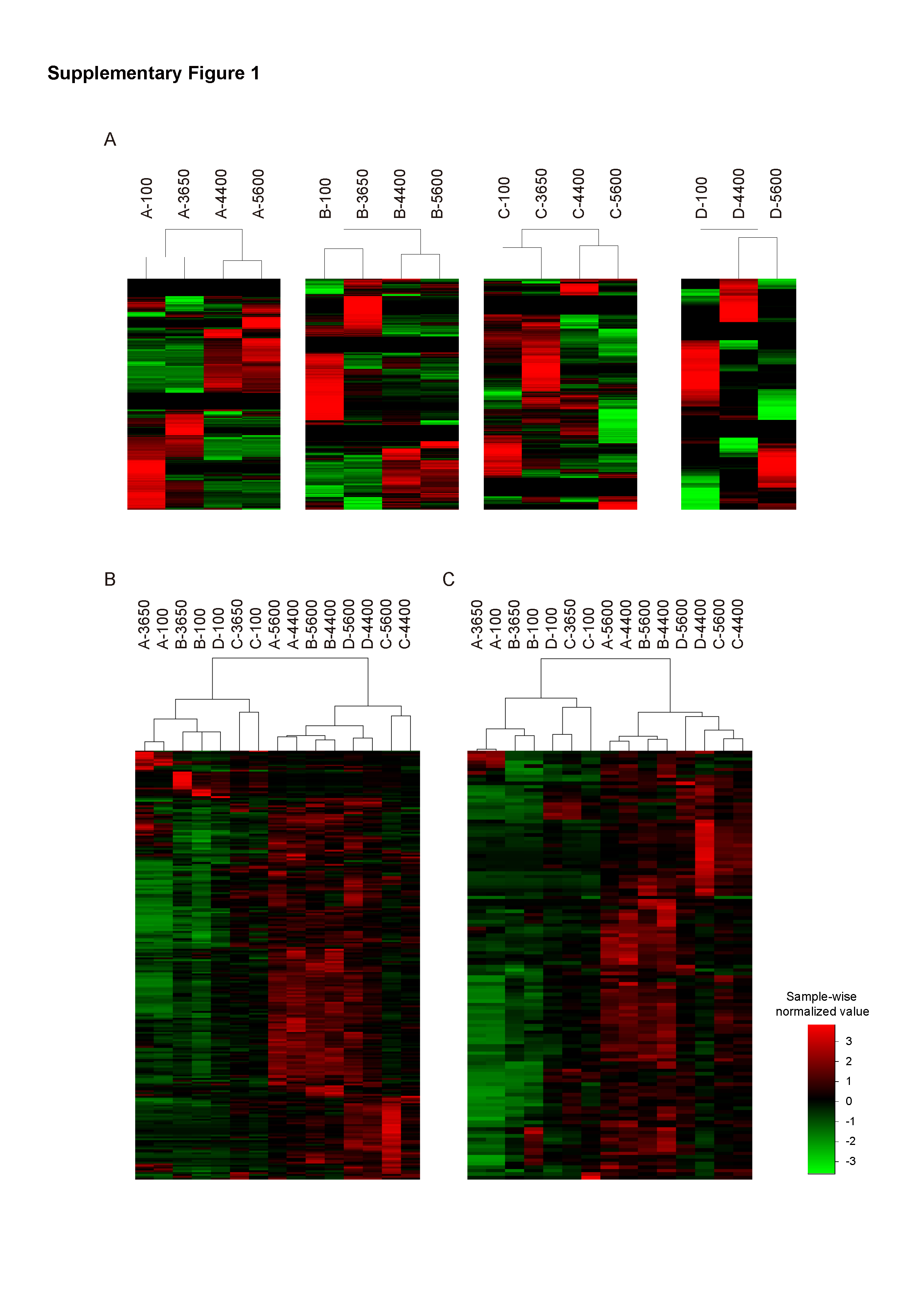

Supplement: Figure S1 — Hierarchical clustering of each individual based on expression profiles of all genes measured by RNA-seq (A). Or the Hierarchical clustering of all samples based on expression profiles of differentially expressed genes (DEGs) selected by comparing 100 m vs. 4,400 m and 100 m vs. 5,600 m (B), or comparing 3,650 m vs. 4,400 m and 5,600 m (C). Samples are named in the format of ‘Climber ID-altitude’. The expression value for each gene is indicated by color intensity, with red representing high expression and green representing low expression. (TIF) [file pone.0031645.s001.tif]

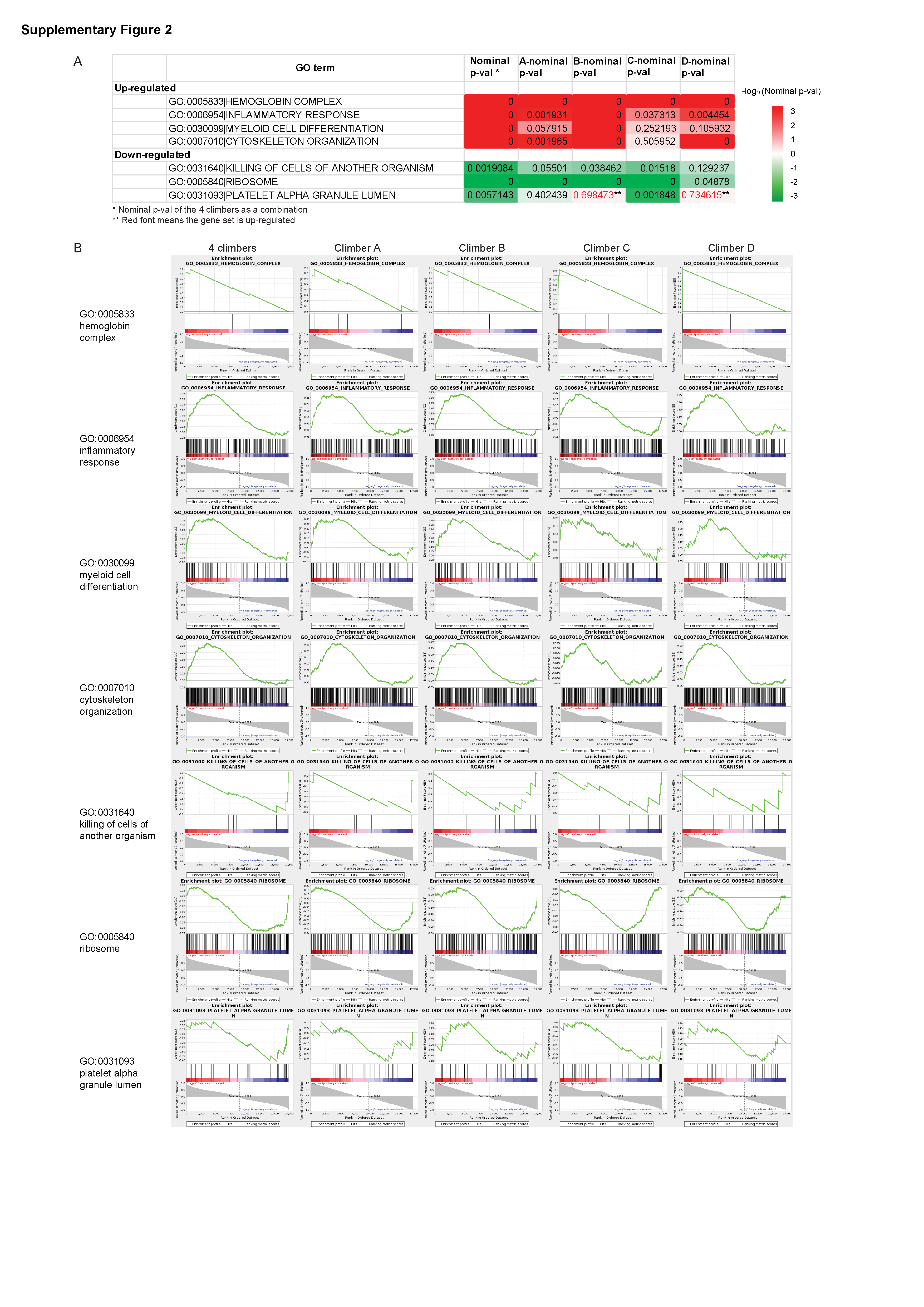

Supplement: Figure S2 — Gene set enrichment analysis (GSEA) summary of seven up- or down- regulated gene sets (A). The expression value represented by -log10(Nominal p-val) for each gene set is indicated by color intensity, with red representing high expression and green representing low expression. And enrichment plots of these gene sets in four climbers are provided as a combination or individually (B). (TIF) [file pone.0031645.s002.tif]

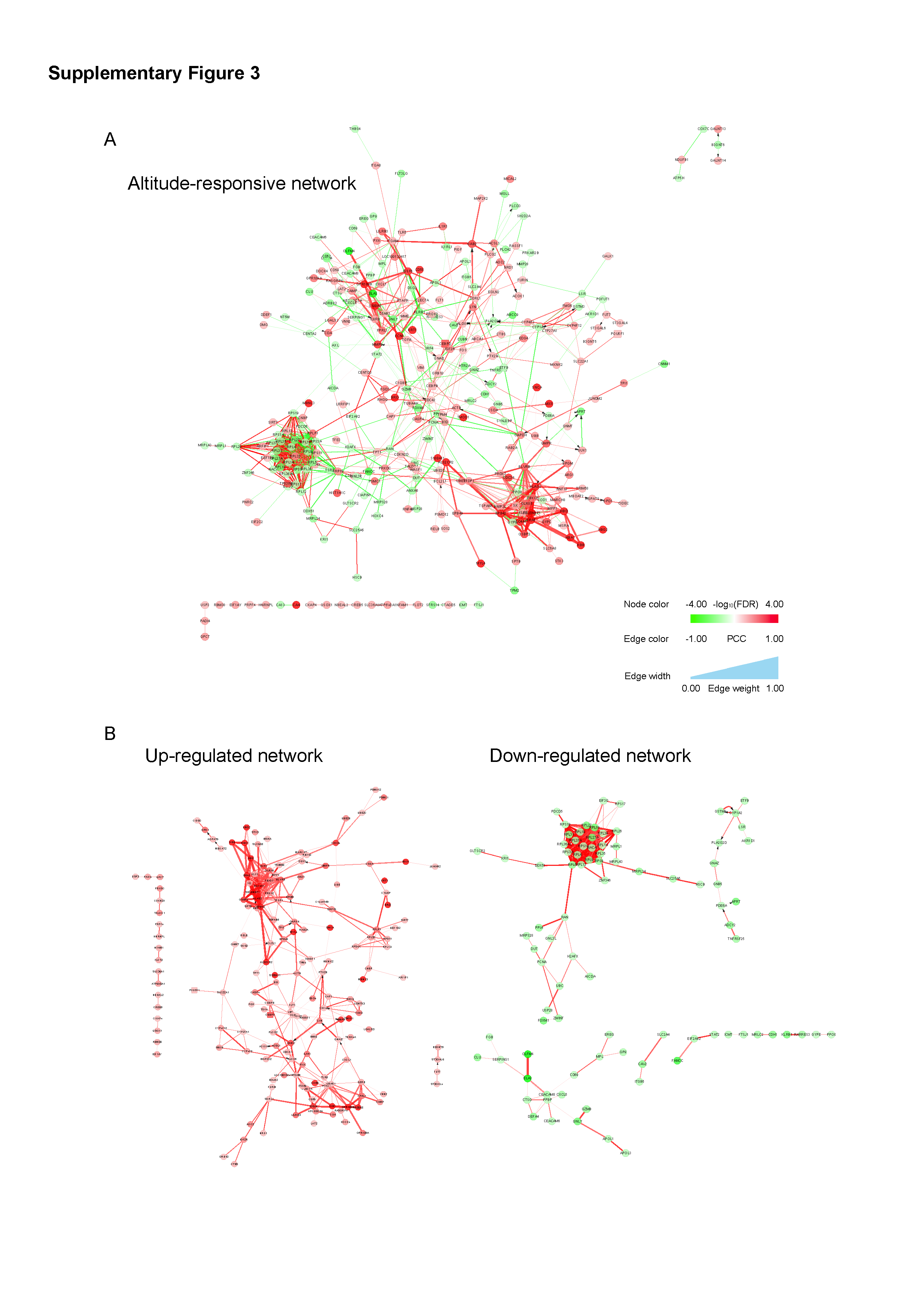

Supplement: Figure S3 — The altitude-responsive network (ARN) (A) as well as the up- or down-regulated sub-network of the ARN (B). (TIF) [file pone.0031645.s003.tif]

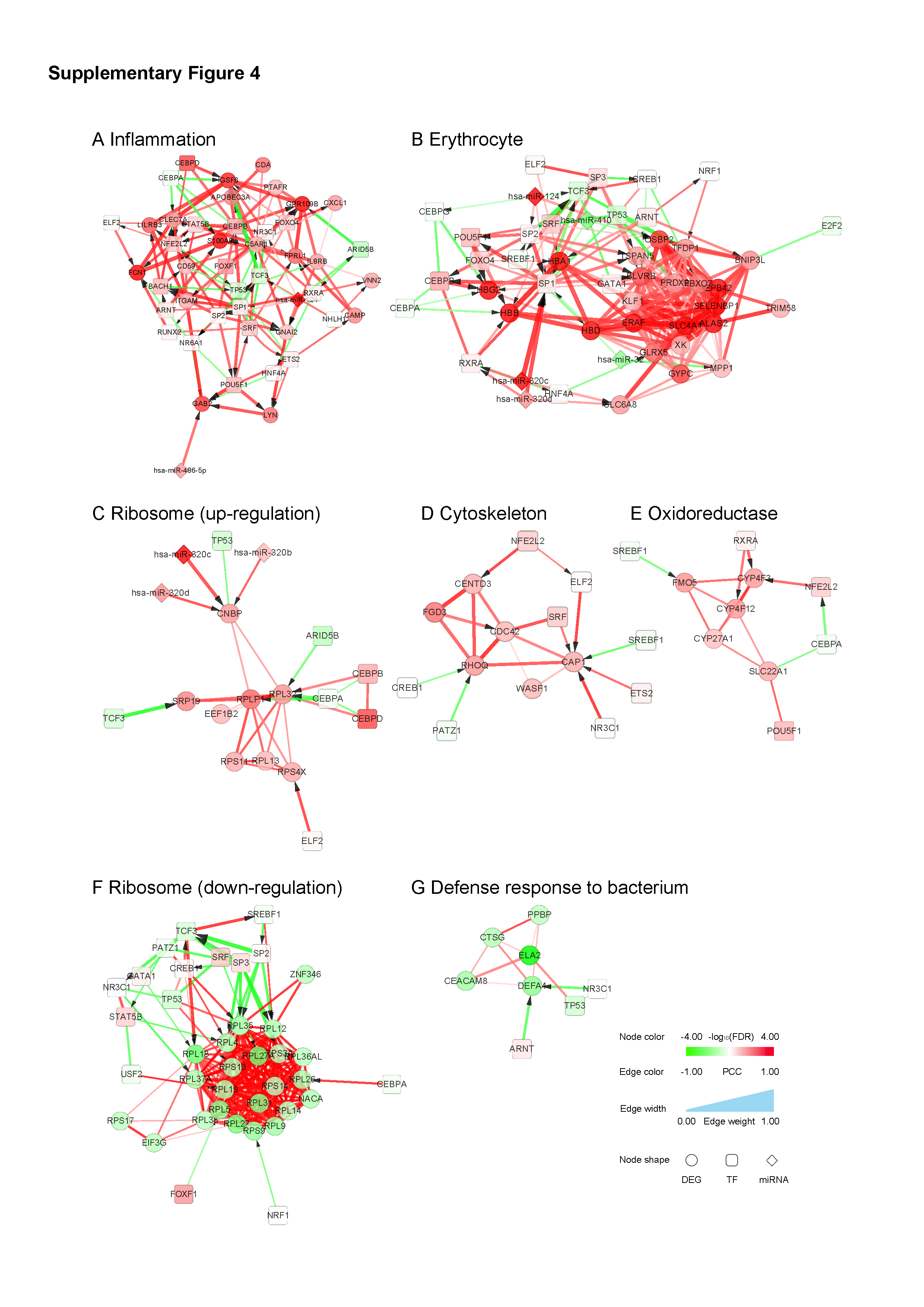

Supplement: Figure S4 — Regulatory networks of the up- or down-regulated network modules. Only the one-step regulatory interactions were included in each module. (TIF) [file pone.0031645.s004.tif]

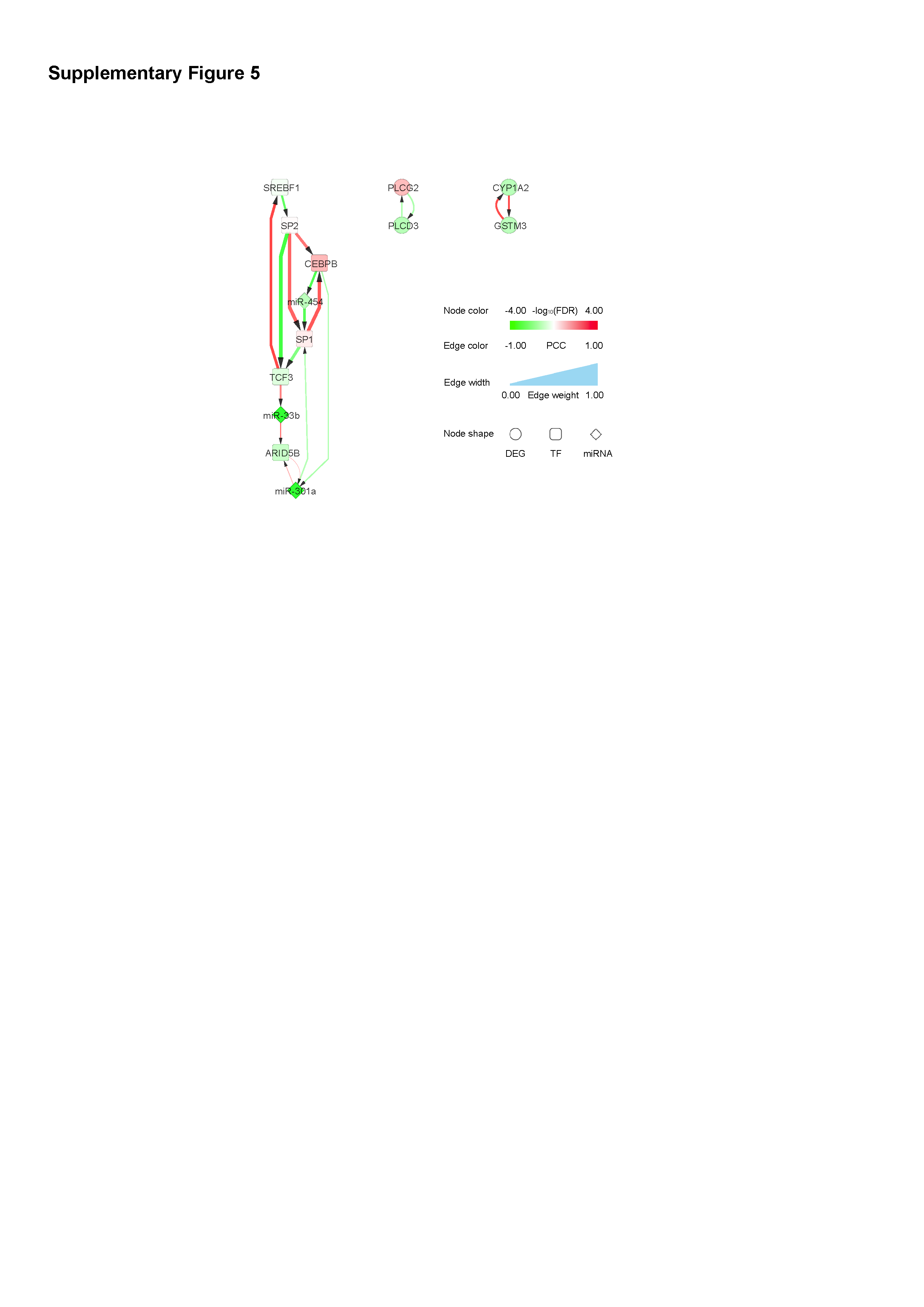

Supplement: Figure S5 — Feedback loops of ≤5 steps found in the full regulatory network. (TIF) [file pone.0031645.s005.tif]

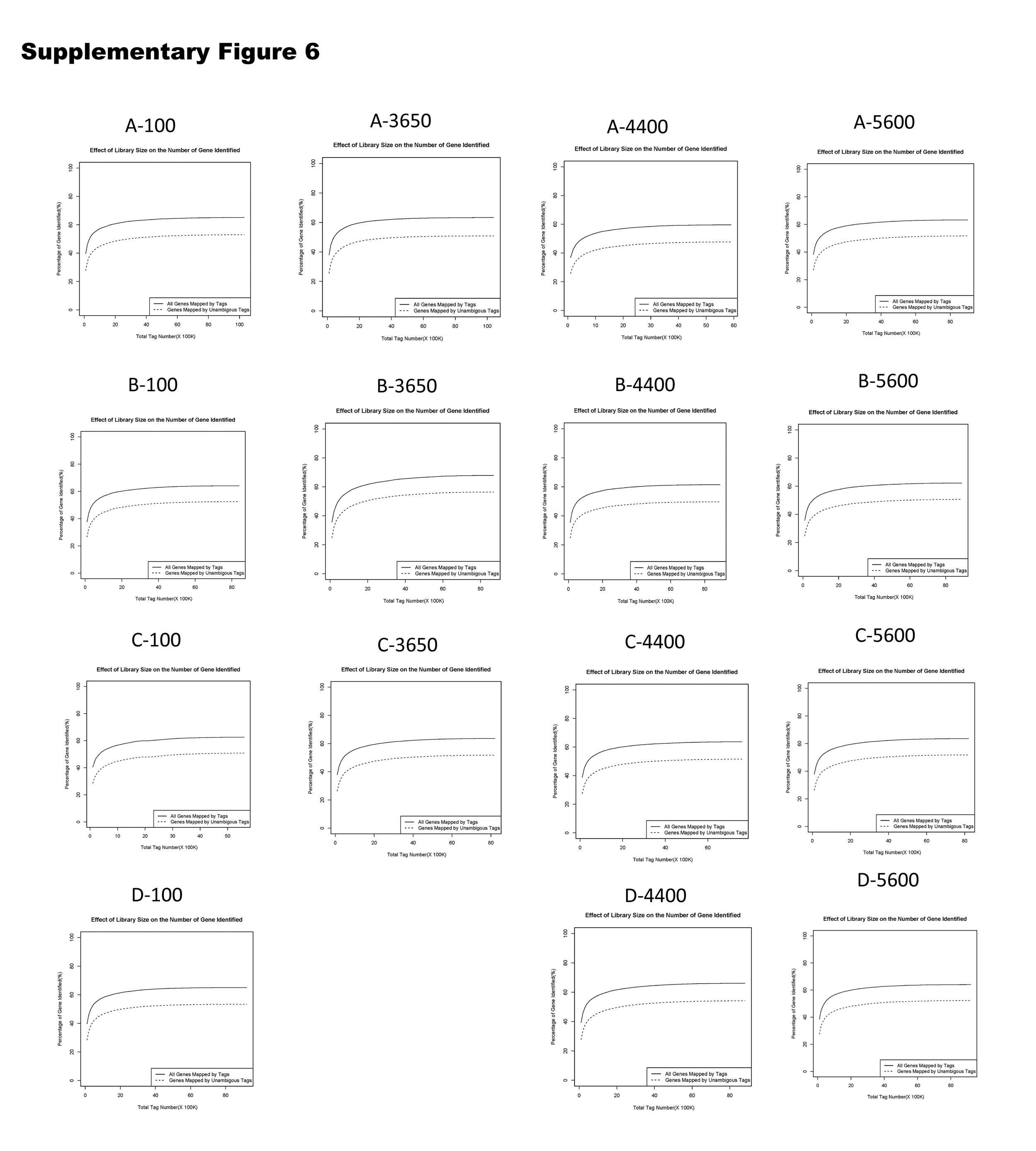

Supplement: Figure S6 — Saturation curves of the samples at the obtained sequencing depth. (TIF) [file pone.0031645.s006.tif]

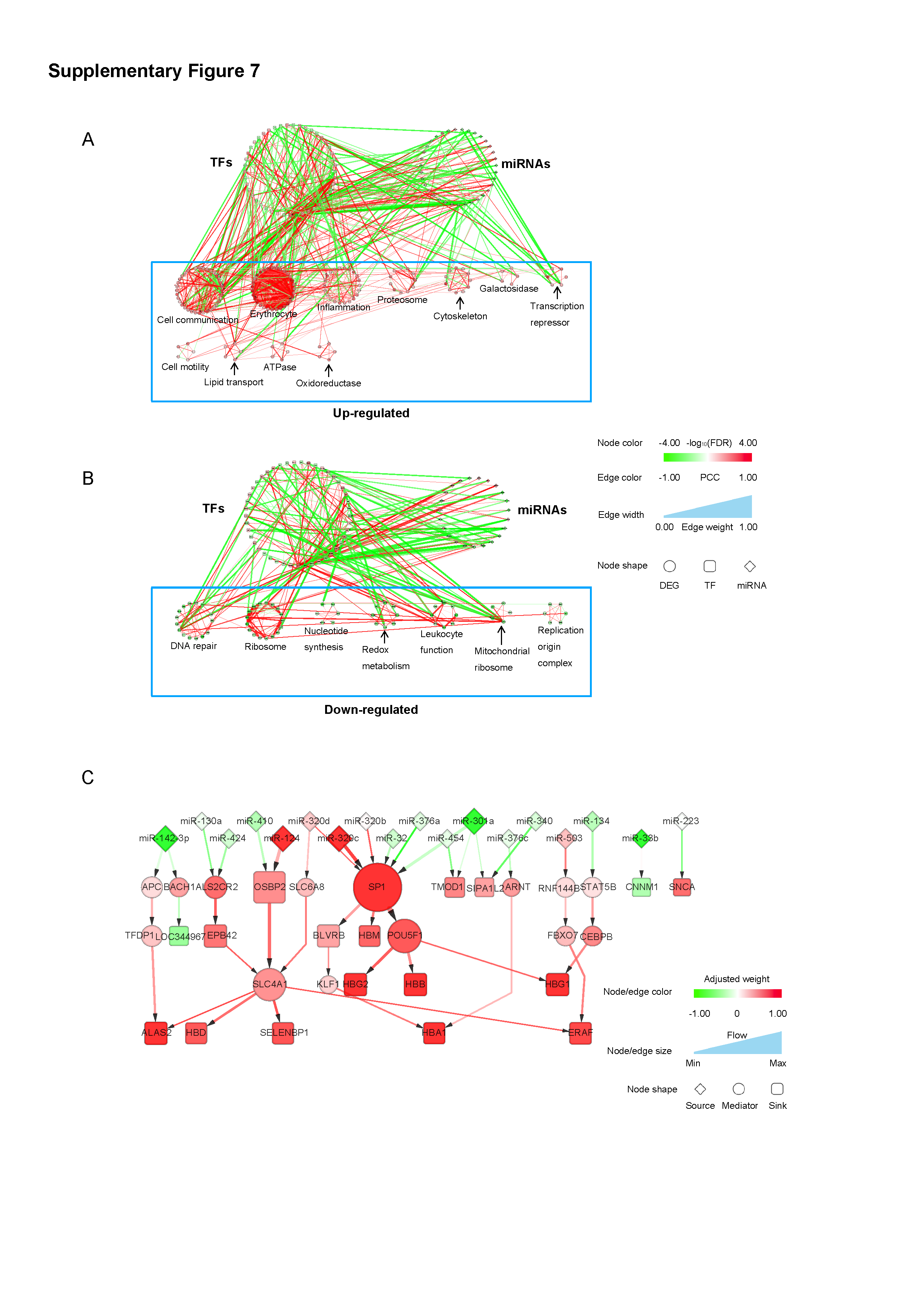

Supplement: Figure S7 — The regulatory modular network for up- (A) or down-regulated (B) genes defined by a less stringent criteria, which is the intersection of at least 2 people with RankProd P-value ≤0.05, and the ResponseNet based on this set of DEGs (C). (TIF) [file pone.0031645.s007.tif]

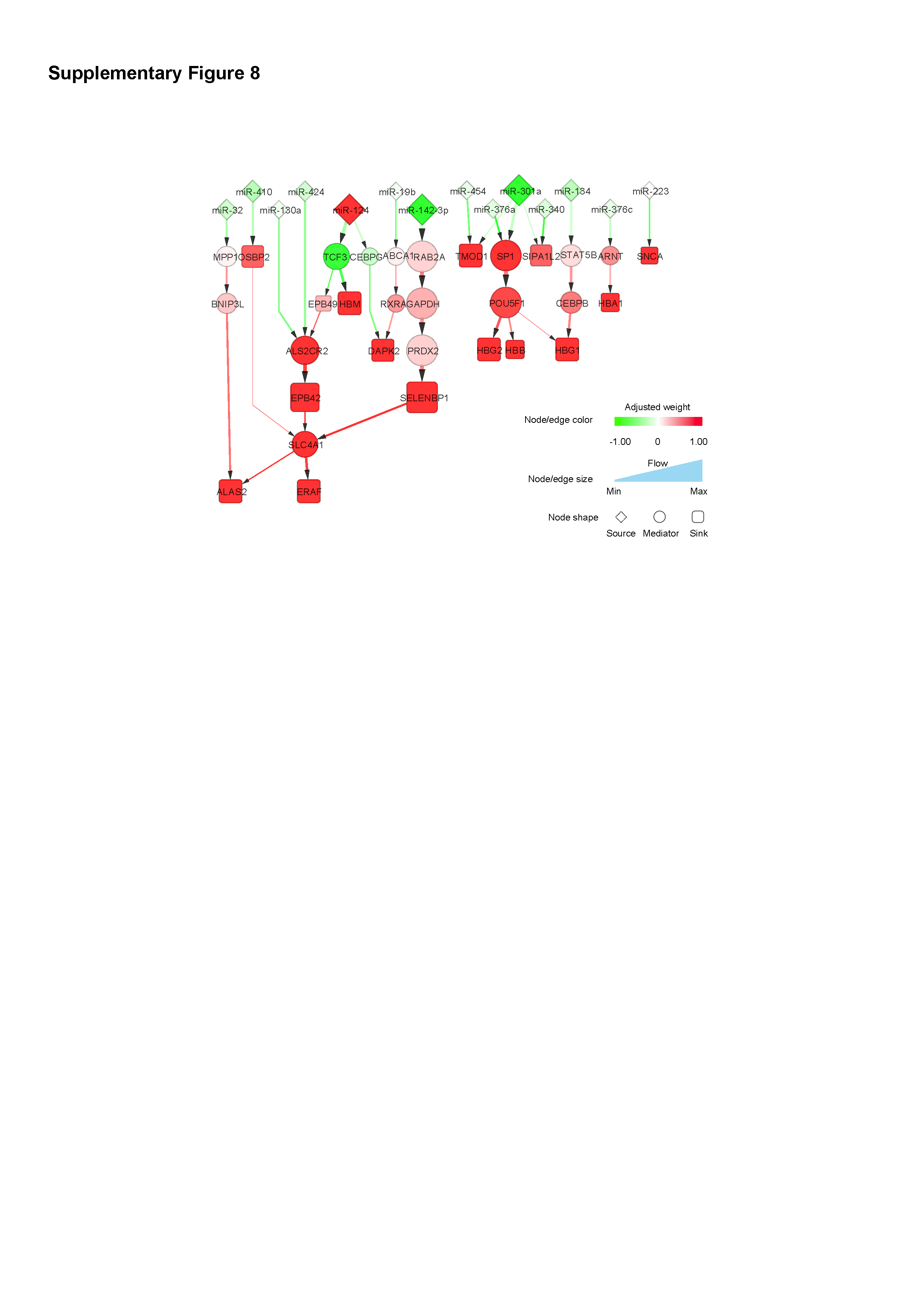

Supplement: Figure S8 — The ResponseNet of retaining only the anti-correlations between miRNAs and targets. All the other settings are exactly the same as Figure 3B. (TIF) [file pone.0031645.s008.tif]
