# Supplementary material for: Transcriptome and Network Changes in Climbers at Extreme Altitudes
Source: PLoS One. 2012 Feb 29;7(2):e31645. doi: 10.1371/journal.pone.0031645 (PMC3290542; doi:10.1371/journal.pone.0031645)
Supplement: Methods S1 — (DOC) [file pone.0031645.s023.doc]

**Supplementary Methods**

**Transcriptome and network changes in climbers at extreme altitudes**

Fang Chen^1,3,5^,*, Wei Zhang^2,4,5^,*, Yu Liang^3,5,＊^, Jialiang Huang^2,4,5^,*, Kui Li^3,5^,*, Christopher D. Green^2^,*, Jiancheng Liu^2,4,5^,*, Guojie Zhang^1^ , Bing Zhou^2,4,5^, Xin Yi^1,3,5^, Wei Wang^1^, Hang Liu^1^, Xiaohong Xu^1^, Feng Shen^1^, Ning Qu^1^, Yading Wang^1^, Guoyi Gao^6^, A San^1,3,5^, Luo Sang Jiang Bai^1^, Hua Sang^1^, Xiangdong Fang^3^, , Karsten Kristiansen^7^, Huanming Yang^1^, Jun Wang^1,7,#^, Jing-Dong J. Han^2,#^ & Jian Wang^1,#^

***RNA sequencing and data processing***

Total RNA was extracted by Trizol reagent and quantified using Agilent Bioanalyzer 2100 with the RNA integrity number greater than 8.0 before Illumina Genome Analyzer (GA) sequencing. We performed RNA sequencing (RNA-seq) using the Illumina Genome Analyzer to measure mRNA expression levels from the 15 blood samples. Typically, 2–4 ug total RNA were used in library construction. Total RNA was reverse transcribed to double-stranded cDNA, the cDNA was digested with NlaIII and ligated to an Illumina specific adapter containing a recognition site of MmeI. Following MmeI digestion, a second Illumina adapter, containing a 2-bp degenerate 3’ overhang was ligated. Tags flanked by both adapters were enriched by an 18-cycle PCR. The PCR products were run on a 12% PAGE gel and the ~85-bp DNA band was excised and purified for cluster generation and sequencing analysis. The sequences were aligned onto human RefSeq database (<ftp://ftp.ncbi.nih.gov/refseq>) using SOAP software . Only uniquely mapped sequences to RefSeq genes were kept for subsequent analysis. For each mRNA sample, we generated on average a total of 8.4 M (ranging from 5.5 M to 10 M) raw tags with 7.9 M (from 4.7 M to 9.9 M) clean tags (sequences without undefined nucleotides and excluding sequences only sequenced once or adaptors sequences). On average, 63% of the reads matched known genes, and ~13,000 genes had expression level data (Table S11, S12). Saturation analysis indicated gene coverage was saturated at the obtained sequencing depth (Figure S6). All libraries were normalized to 1 M sequences according to clean tags.

The expression of miRNA from 14 samples (the Lhasa climber C blood sample was insufficient for sequencing) were also measured using the Illumina GA platform. Small RNA molecules under 30 bases were purified from 4–10ug total RNA by electrophoresis on a 12% PAGE gel and ligated with a pair of Illumina adaptors to their 5’ and 3’ ends, the small RNA molecules were reverse transcribed and amplified using the adaptor primers for 18 cycles and fragments of ~90 bp (small RNA+adaptors) were isolated from PAGE gel. Purified DNAs were used directly for cluster generation and sequencing analysis using the Illumina GA according to the manufacturer’s instructions. We generated about 8 M raw reads for each library and over 60% of the reads could be aligned to miRNAs from miRBase (<http://microrna.sanger.ac.uk/sequences/index.shtml>). Finally, up to 300–400 miRNA could be identified for each library (Table S13, S14). All miRNA libraries were normalized to a total of 1 M reads per sample according to clean tags.

***Identifying differential expression by RankProd***

We used the RankProd to identify the differential expression between two extremely high (4,400 m and 5,600 m) and two low altitudes (100 m and 3650 m). Given the large individual variations among the limited number of samples, we performed the RankProd analysis by treating the four individuals as from four different origins. This approach used a build-in function RPadvance in the R package RankProd. When running the RPadvance program, within each individual, the data of both two low altitudes were compared with both two high altitudes. Thus for each gene the four individuals’ fold changes were separately calculated by comparing 100m vs. 4,400m, 100m vs. 5,600m, 3,650m vs. 4,400m, and 3,650m vs. 5,600m. Then the relative ranks of these fold changes (along different genes) were used to calculate a geometric mean for each gene, which is the so called Rank Product (RP) value. After that 100 permutations were performed by shuffling the genes’ values in each data point (altitude) in order to calculate the *P*-values of the genes’ RP values. Then, we used Benjamini-Hochberg corredted FDR ≤ 0.1 as a cutoff to define the DEGs. More details could be found in the original paper of RankProd . We also applied the same method to identify differentially expressed miRNA. As RankProd is designed to robustly analyze small size samples with a few repeats, our four biological samples are more than enough to generate robust statistics by RankProd.

***Definition of the interactions***

The functional interactions was defined as the union of literature curated interactions from KEGG (<http://www.genome.jp/kegg/>) and the high confidence interactions predicted by heterogeneous evidences from STRING (Confidence Score > 0.7) and IntNetDB (Likelihood Ratio > 7), downloaded on April 9, 2010.

A TF->target interaction was predicted when the target gene (or miRNA) was found to contain the consensus binding motif of the TF within the 1 Kb sequence upstream of the transcription start site (TSS) (obtained from UCSC hg18) using the STORM software at *P* ≤ 10^-5^. The motifs were determined by the position weight matrix of the TFs in the JASPAR or TRANSFAC database. Sequences 1 Kb upstream of 1000 randomly selected genes were used as a background.

Potential miRNA->target interactions were downloaded from PicTar and TargetScan databases on April 18, 2009. We used the union of the interactions from PicTar and TargetScan for the further analysis.

The functional interactions were used as edges to construct the altitude responsive network (ARN). And the latter two types of interactions were added to the ARN to construct the full regulatory network (FRN).

***Weighting the genes, TFs and miRNAs***

Differentially expressed genes (DEGs) were identified by the RankProd using a cutoff of Benjamini-Hochberg corrected *FDR* ≤ 0.1. We then defined a gene’s weight as *W_gene_* = -log_10_(*FDR*).

The differentially expressed miRNAs were identified using the same method and the same cutoff as the genes, and weighted similarly as *W_miRNA_* = -log_10_(*FDR*). Totally, 16 and 22 significantly up- and down-regulated miRNAs were idnetified, respectively, and used for further analysis.

For weighting the TFs, we first scored each possible TF->target interaction by the Pearson correlation coefficient (PCC) (from 15 data points) multiplied by the target’s weight as *S_TF->target_* = |*PCC*|× *W_gene/miRNA_*, and filtered them by empirical *P* ≤ 0.01 (i.e., at the top 1% among 10^6^ random TF->gene/miRNA pairs regardless of whether binding sites are present or not). (Please note that the score mentioned here is only used in this step to filter the TF->target interactions and is NOT the edge weight of TF->target interactions. And TF->target interactions were only filtered once using this criterion.) Then by restricting the targets to the 723 DEGs, we weighted the TFs to reflect their prestige centrality , which was scored by the sum of the products of PCC between the TF and each of its targets and the target’s node weight. To prevent the overestimation of the out degree, we corrected it by log_10_(*k*)/*k*, where *k* is the target number of the TF. The TF weighting formula is $W_{TF}=\frac{\log_{10}(k)}{k}\sum_{i=1}^{k} (|PCC_{i}|\times W_{i})$. After that top 34 (approximately 10%) weighted TFs were defined as hub TFs and used for further analysis.

To make the weights of hub TFs directly comparable with the DEGs and differentially expressed miRNAs, we scaled the weights to a range between 0 and 1 by *W_i_’* = (*W_i_* - *W_min_*) / (*W_max_* - *W_min_*) within the 723 DEGs, 38 differentially expressed miRNAs, and 34 hub TFs.

The DEGs, differentially expressed miRNAs, and hub TFs were used as nodes to construct networks.

***Weighting the interactions***

We developed an edge weighting formula similar to a previously published one to integrate two sources of information, 1) the significance of the two interacting genes’ differential expression and 2) the correlation between the two genes, into a single measurement. Thus, an edge weight is defined as the mean scaled weights of the two nodes connected by the edge multiplied by the PCC (from 10 data points for miRNAs and 15 for mRNAs) between the expression profiles of the two nodes (DEGs, hub TFs or differentially expressed miRNAs) as *W_edge_=* |*PCC_ij_*|(*W’_i_* + *W’_j_*) / 2.

The functional interactions and miRNA->target interactions were filtered by *W_edge_* using a cutoff of empirical *P* ≤ 0.1 or 0.01 based on the 10^6^ random pairing between all genes or between all miRNAs and all genes, respectively. The TF->targets interactions were filtered as we described when weighting the TFs.

The rational of using a more stringent cutoff (*P* ≤ 0.01) for TF->targets and miRNA->targets is that these interactions were merely predicted by the binding motifs on the target sequences, therefore they are prone to give rise to false positives and of much lower confidence than the functional interactions which were literature-curated or predicted by various evidences.

***Extracting ResponseNet by network flow***

ResponseNet is a flow optimization algorithm that searches for pathways which carry the flow from a set of source nodes to a set of target nodes in the network at the minimal cost. It was originally designed to search for the most important pathways in a large possible network, linking genes required for a biological response identified by genetic screens to genes whose expression significantly changed during the response. Here we do not have the genetically identified regulators, so instead we used miRNAs as the source nodes, while treating the genes which were significantly up- or down-regulated in all four climbers (*P* ≤ 0.05) as sink nodes.

Using the edges in the full regulatory network, the ResponseNet with the lowest cost were found by implementing an algorithm described by Yeger-Lotem *et al.*  with the parameter g set to 4.4. The cost of an edge is defined as –log(*W_edge_*).

***Rationales of combining the two low altitude data***

The rationale for the combination of two low altitude data (100 m and 3,650 m) and two high altitude data (4,400 m and 5,600 m) was based on the results of the principal component analysis (PCA) (Fig. 1B). The PCA was performed using all genes detected by RNA-seq, and it shows that at 100 m and 3,650 m the expression profiles exhibit large variations among different individuals but at 4,400 m and 5,600 m the expression profiles of different individuals look quite similar. It suggests that the expression profiles of the 2 high altitudes (4,400 m and 5,600 m) are very different from 100 m and/or 3,650 m although 3,650 m were typically defined as high-altitude and the partial pressure of oxygen is different from 100 m and similar with 4,400 m and 5,600 m. There might be at least two explanations for this: first is the lack of acclimation time at 3,650 m (1 day), second is that at 4,400 m and 5,600 m the climbers had intense physical activities (climbing) while at 100 m or 3,650 m they did not, and it is well known that intense physical activity exacerbate high altitude response.

To further demonstrate that our 3,650 m data should be categorized as low altitude with that of 100 m, we performed hierarchical clustering of the 4 individuals in combination (Figure 1C) or separately (Figure S1A) using all genes detected by RNA-seq. We can see in either case the samples were automatically grouped into two clusters corresponding to low altitudes (100 m and 3,650 m) and two high altitude (4,400 m and 5,600 m), respectively, even before differentially expressed genes (DEGs) were selected.

We also tried to select DEGs using RankProd by only comparing 100 m vs. 4,400 m and 100 m vs. 5,600 m (FDR≤ 0.05) and then perform hierarchical clustering to see whether 3,650 m would still be grouped with 100 m (Figure S1B). And we see that 100 m and 3,650 m were still grouped together even the 3,650 m data was not used in the DEG selection. We also tried to select DEGs by only comparing 3,650 m vs. 4,400 m and 5,600 m (Figure S1C) and got a very similar result. Finally, we tried to find DEGs between 100 m and 3,650 m, but there are only 3 and 7 DEGs passed the FDR ≤ 0.05 and FDR ≤ 0.1 cutoff, respectively. Again, this indicates that the expression profiles at 100 m and 3,650 m are very similar.

***Details for t-test and ANOVA approaches***

We tried to use t-test or ANOVA to identify the differential expression in response to extreme altitude.

For t-test, if using Student's original definition of the t-test, the two populations being compared should have the same variance. So we first performed F-test and found that 4,787 out of 17,365 genes (27.6%) detected by RNA-seq have different variance between the low and high altitude groups (F-test *P*-value ≤ 0.05). Therefore we used the Welch's t-test, which is an adaptation of Student’s t-test intended for use with two samples having possibly unequal variances. The formula is

$$\text{t}=\frac{\overline{X_{1}} - \overline{X_{2}}}{\sqrt{\frac{\text{s}_{1}^{2}}{\text{N}_{1}}+\frac{\text{s}_{2}^{2}}{\text{N}_{2}}}}$$

We performed the Welch’s t-test on all 17,365 genes detected by RNA-seq and 1,586 of them passed the cutoff of *P*-value≤0.05. However, after the Benjamini-Hochberg multiple testing correction, only one gene passed the cutoff of FDR≤0.1. Thus, the main reason we did not use t-test is that only one differentially expressed gene was found.

We also tried to perform log transformation and/or quantile normalization before performing t-test, but the results are similar (always find only one differentially expressed gene). Then we tried paired t-test by randomly matching the two high and two low altitudes (4,400 m/5,600 m ~ 100 m/3,650 m) for each individual in order to reduce the variance between individuals. But we did not find any differentially expressed gene by this approach.

Furthermore, an assumption of the t-test is that each of the population being compared should follow a normal distribution. So we performed the Kolmogorov-Smirnov test and found none of the genes followed normal distribution in both low and high altitude groups (K-S test *P*-value>0.05). So theoretically our data are not suitable for performing t-test.

For one-way ANOVA the situation is similar, 11,430 out of 17,365 genes (65.8%) did not follow normal distribution (K-S test *P*-value≤ 0.05), and 6,138 out of 17,365 genes (35.3%) do not have the same variance between the 4 altitudes. So theoretically our data do not follow these 2 assumptions of the ANOVA, thus they are not suitable for ANOVA. And practically, none of the genes could pass the cutoff of FDR≤0.1.

Thus, we finally chose a nonparametric method RankProd which does not have such assumptions under the model and is robust when dealing with noisy data with large between-replicate variances. Using RankProd, 723 and 392 genes passed the cutoff of FDR≤0.1 and 0.05, respectively.

***Topological analysis of the altitude responsive network (ARN)***

Average node degree, clustering coefficient, and diameter of the ARN and the up- and the down-regulated networks are calculated by NetworkAnalyzer, a plug-in of the Cytoscape. As a control, ten random networks were generated by randomly select 723 genes, which is the number of all the DEGs in response to the extreme altitude, in our full gene list detected by RNA-seq, and then map them to the functional network as we illustrated in the results section. We found that the ARN has a smaller diameter than those of random networks; and the ARN, and the up- and down-regulated networks generally have higher clustering coefficient and average node degree than those of the random networks (Table S5). These findings indicated that there are obvious existence of network modules in the ARN, and the up- and down-regulated networks.

***Toggle switches as homeostasis regulators***

The well-coordinated up- and down-regulations of basic cellular processes and cell fates (Figure 2A and B) reveals the system’s homeostatic capability. Feedback loops, especially toggle switches (feedback loops composed of mutually inhibitory interactions), are often crucial in the maintenance of homeostasis of a system. In the FRN, we found three feedback modules (connected sub-graphs that consisted of feedback loops of ≤ 5 steps). Considering a regulatory interaction between two nodes that have opposite expression patterns as inhibitory interactions, we identified two conspicuous toggle switch modules: one is a complicated module between miRNAs and TFs containing several toggle switches among SP1/2, CEBPB, ARID5B, TCF3, SREBF1, and miR-454/-301a/33b; and the other is a simple toggle switch between PLCG2 and PLCD3, representing the metabolic pathway of inositol phosphate metabolism (Figure S5). The third feedback module marked coordinate down-regulation of two genes involved in removing reactive oxygen species (ROS): glutathione transferase (GSTM3) and P450 oxidoreductases (CYP1A2) (Figure S5).

The ResponseNet analysis captured the important regulatory nodes in the toggle switches. Four out of seven (57.1%) genes in the miRNA mediated toggle switches were included in the ResponseNet, whereas none out of the four genes in other types of toggle switches were included

***Regulatory networks based on the less stringent definition of DEGs***

In order to construct larger modular and regulatory networks, we tried to use a less stringent definition of DEGs, which is the intersection of genes that passed the significance level of RankProd *P*-value (instead of FDR) ≤ 0.05 in at least two climbers, and yielded 1,528 DEGs in total. Then each DEG is weighted by *-*Σlog*P_i_*, where *P_i_* is the RankProd *P*-value in each of the four climbers.

Using the same methods as we described in the main text, we constructed the ARN and delineated the network modules. By examining enriched GO terms and KEGG pathways using the Fisher’s exact test, we found that the up-regulated network contained 11 modules corresponding to the functions ‘cell communications’, ‘erythrocyte development/oxygen binding’, ‘inflammation’, ‘proteosome/kinetochore’, ‘cytoskeleton’, ‘cell motility’, ‘lipid transport’, ‘ATPase complex’, ‘transcription repressor’, ‘oxidoreductase’ and ‘galactosidase’, respectively. In the down-regulated network, we found seven modules that were enriched for the functions ‘DNA replication origin complex’, ‘nucleotide synthesis’, ‘DNA repair/cell cycle’, ‘ribosome’, ‘mitochondrial ribosome’, ‘Redox metabolism’, and ‘leukocyte (T, B and killer cells) functions’, respectively (Figure S7A and B).

To identify transcriptional regulation of altitude response, the TFs and miRNAs were filtered and weighted using the same methods and the ResponseNet was similarly performed as in the main text. Although this regulatory network was distilled based on a template of twice as many DEGs as in the main text (1,528 versus 723), the most important regulators and pathways found by them are largely the same (Figure S7C compared with Figure 3B). In fact, 81.6% of the nodes in this ResponseNet (40 out of 49 in Figure S7C) were overlapped with the one shown in the Figure 3B (55 nodes), including the important regulators we discussed in the main text such as SP1, OCT4 (POU5F1), HIF1B (ARNT), and BACH1. It further demonstrated the advantage of the network integration approach compared to the conventional single step differential gene expression detection approach.

***Retaining only the anti-correlated interactions between miRNAs and their targets in the ResponseNet approach***

Although there might exist both “coordinate” and “compensatory” relationships between miRNAs and their targets in response of an external stimulus such as extreme altitude. The anti-correlated miRNA-target interactions would still possibly be of more confidence than the correlated ones. We therefore tried to retain only the anti-correlations between miRNA and targets in the ResponseNet approach. As a result (Figure S8) the ResponseNet is similar as the one shown in the manuscript (Figure 3B). We could still see OCT4 as an important regulator linking SP1 and HBG1/2 and HBB.

***Checking the expression of erythropoietin at extreme altitude.***

One of the known physiological responses to high altitude is erythrocyte expansion. Induction of erythropoietin by HIF1 has been proposed to be responsible for such changes at extreme altitude, although no adaptive changes have been found in the erythropoietin gene in the Andeans, a population well adapted to high altitude . We also did not see an increase in erythropoietin expression, but this was not unexpected given that erythropoietin is not produced in blood cells . The tissue atlas also revealed that erythropoietin mRNA is specifically expressed in several cell and tissue types, while blood cells have only background expression level. We did find changes in other major players in the erythropoietin pathways, PLCg2 (PLCG2), FOS, SOS2, RELB, PPP2R3A, and MAP2K2, in our high-altitude induced genes. However, the concomitant induction of a few other pathways, such as the G-protein coupled receptor protein signaling pathway, Toll-like receptor signaling pathway, and apoptosis pathway, indicate that the erythropoietin pathway, despite being a major signaling pathway activated, does not explain all the gene expression changes. Interestingly, we found that none of the highly weighted transcription factors, OCT4, SP1/2/3, TCF3, CEBPB and HIF1B (ARNT), belong to the erythropoietin pathway, even though some of them, like HIF1B, could work upstream of the pathway. Among the highly weighted transcription factors, SP1/3, CEBPB and HIF1B (ARNT) have been shown to mediate a hypoxia response in various types of cells. Their primary mode of action, however, is not through direct regulation of red blood cell functions. OCT4, on the other hand, was predicted by our network analysis to be a key regulator mediating the high-altitude response and to directly regulate hemoglobin genes.

**REFERENCES**

1. Li R, Li Y, Kristiansen K, Wang J (2008) SOAP: short oligonucleotide alignment program. Bioinformatics 24: 713-714.

2. Hong F, Breitling R, McEntee CW, Wittner BS, Nemhauser JL, et al. (2006) RankProd: a bioconductor package for detecting differentially expressed genes in meta-analysis. Bioinformatics 22: 2825-2827.

3. Jensen LJ, Kuhn M, Stark M, Chaffron S, Creevey C, et al. (2009) STRING 8--a global view on proteins and their functional interactions in 630 organisms. Nucleic Acids Res 37: D412-416.

4. Xia K, Dong D, Han JD (2006) IntNetDB v1.0: an integrated protein-protein interaction network database generated by a probabilistic model. BMC Bioinformatics 7: 508.

5. Smith AD, Sumazin P, Xuan Z, Zhang MQ (2006) DNA motifs in human and mouse proximal promoters predict tissue-specific expression. Proc Natl Acad Sci U S A 103: 6275-6280.

6. Bryne JC, Valen E, Tang MH, Marstrand T, Winther O, et al. (2008) JASPAR, the open access database of transcription factor-binding profiles: new content and tools in the 2008 update. Nucleic Acids Res 36: D102-106.

7. Matys V, Fricke E, Geffers R, Gossling E, Haubrock M, et al. (2003) TRANSFAC: transcriptional regulation, from patterns to profiles. Nucleic Acids Res 31: 374-378.

8. Lall S, Grun D, Krek A, Chen K, Wang YL, et al. (2006) A genome-wide map of conserved microRNA targets in C. elegans. Curr Biol 16: 460-471.

9. Friedman RC, Farh KK, Burge CB, Bartel DP (2009) Most mammalian mRNAs are conserved targets of microRNAs. Genome Res 19: 92-105.

10. Torkamani A, Schork NJ (2009) Prestige centrality-based functional outlier detection in gene expression analysis. Bioinformatics 25: 2222-2228.

11. Reverter A, Hudson NJ, Nagaraj SH, Perez-Enciso M, Dalrymple BP Regulatory impact factors: unraveling the transcriptional regulation of complex traits from expression data. Bioinformatics 26: 896-904.

12. Yeger-Lotem E, Riva L, Su LJ, Gitler AD, Cashikar AG, et al. (2009) Bridging high-throughput genetic and transcriptional data reveals cellular responses to alpha-synuclein toxicity. Nat Genet 41: 316-323.

13. Assenov Y, Ramirez F, Schelhorn SE, Lengauer T, Albrecht M (2008) Computing topological parameters of biological networks. Bioinformatics 24: 282-284.

14. Smoot ME, Ono K, Ruscheinski J, Wang PL, Ideker T Cytoscape 2.8: new features for data integration and network visualization. Bioinformatics 27: 431-432.

15. Hasty J, McMillen D, Collins JJ (2002) Engineered gene circuits. Nature 420: 224-230.

16. Hochachka PW, Rupert JL (2003) Fine tuning the HIF-1 'global' O2 sensor for hypobaric hypoxia in Andean high-altitude natives. Bioessays 25: 515-519.

17. Lacombe C, Da Silva JL, Bruneval P, Casadevall N, Camilleri JP, et al. (1991) Erythropoietin: sites of synthesis and regulation of secretion. Am J Kidney Dis 18: 14-19.

18. Su AI, Wiltshire T, Batalov S, Lapp H, Ching KA, et al. (2004) A gene atlas of the mouse and human protein-encoding transcriptomes. Proc Natl Acad Sci U S A 101: 6062-6067.
